# Antimicrobial Activity of Monoramnholipids Produced by Bacterial Strains Isolated from the Ross Sea (Antarctica) †

**DOI:** 10.3390/md14050083

**Published:** 2016-04-26

**Authors:** Pietro Tedesco, Isabel Maida, Fortunato Palma Esposito, Emiliana Tortorella, Karolina Subko, Chidinma Christiana Ezeofor, Ying Zhang, Jioji Tabudravu, Marcel Jaspars, Renato Fani, Donatella de Pascale

**Affiliations:** 1 Institute of Protein Biochemistry, National Research Council, Via P. Castellino, 111, I-80131 Naples, Italy; p.tedesco@ibp.cnr.it (P.T.); f.palma@ibp.cnr.it (F.P.E.); e.tortorella@ibp.cnr.it (E.T.); 2 Department of Biology, University of Florence, Via Madonna del Piano 6, I-50019 Sesto Fiorentino (FI), Italy; isabel.maida@unifi.it (I.M.); renato.fani@unifi.it (R.F.); 3 Marine Biodiscovery Centre, Department of Chemistry, University of Aberdeen, Old Aberdeen, AB24 3UE Scotland, UK; k.subko.11@aberdeen.ac.uk (K.S.); chidinma.christiana.ezeofor.14@aberdeen.ac.uk (C.C.E.); ying.zhang.14@aberdeen.ac.uk (Y.Z.); j.tabudravu@abdn.ac.uk (J.T.); m.jaspars@abdn.ac.uk (M.J.)

**Keywords:** antimicrobials, ramnholipids, Antarctic, Bcc, microorganisms

## Abstract

Microorganisms living in extreme environments represent a huge reservoir of novel antimicrobial compounds and possibly of novel chemical families. Antarctica is one of the most extraordinary places on Earth and exhibits many distinctive features. Antarctic microorganisms are well known producers of valuable secondary metabolites. Specifically, several Antarctic strains have been reported to inhibit opportunistic human pathogens strains belonging to *Burkholderia cepacia* complex (Bcc). Herein, we applied a biodiscovery pipeline for the identification of anti-Bcc compounds. Antarctic sub-sea sediments were collected from the Ross Sea, and used to isolate 25 microorganisms, which were phylogenetically affiliated to three bacterial genera (*Psychrobacter*, *Arthrobacter*, and *Pseudomonas*) via sequencing and analysis of 16S rRNA genes. They were then subjected to a primary cell-based screening to determine their bioactivity against Bcc strains. Positive isolates were used to produce crude extracts from microbial spent culture media, to perform the secondary screening. Strain *Pseudomonas* BNT1 was then selected for bioassay-guided purification employing SPE and HPLC. Finally, LC-MS and NMR structurally resolved the purified bioactive compounds. With this strategy, we achieved the isolation of three rhamnolipids, two of which were new, endowed with high (MIC < 1 μg/mL) and unreported antimicrobial activity against Bcc strains.

## 1. Introduction

The alarming rise of Multi-Drug Resistance (MDR) bacteria in the last few decades has highlighted the need for novel antimicrobial compounds and for effective drug discovery approaches [1,2]. Natural products are the largest source of new antibiotic molecules, representing about two-thirds of new antibacterial therapies approved between 1980 and 2010 [3,4]. Bioprospecting for natural products from unexplored natural environments, such as the marine environment is considered a promising strategy to identify novel compounds. It is increasingly recognized that a huge number of natural products and novel chemical entities exist in these environments, but the overwhelming biological diversity of these environments has so far only been explored to a very limited extent [5,6]. The Antarctic environment, as well as having incredibly low temperatures, possesses other diverse traits that may have helped to shape the unique way in which Antarctic bacteria have evolved. This extreme environment contains hyper-salinity that exists in sea ice brine channels, a lack of free water due to freezing temperatures, as well as low nutrient availability. Unique light conditions also exist due to the high latitude of the region. Several studies have shown that Antarctic bacteria harvested from Antarctic corals and sponges are a promising source of new antimicrobial compounds [7,8,9,10,11,12,13,14]. Specifically, several Antarctic strains belonging to the genus *Pseudoalteromonas*, *Psychrobacter*, *Pseudomonas*, and *Arthrobacter*, were able to inhibit the growth of several strains belonging to the *Burkholderia cepacia* complex (Bcc) [11,14]. Further studies demonstrated that the antimicrobial activity relies (at least in part) on the production of Volatile Organic Compounds (VOCs) [12,13,15]. The Bcc consists of at least 20 closely related species inhabiting different ecological niches such as water, soil, plants rizosphere, and plants and animals [16,17,18]. Bcc are also opportunistic human pathogens that cause lung infections in immune-compromised individuals, including cystic fibrosis (CF) patients [19]. In one-third of infected individuals, it causes the “cepacia syndrome”—a form of septic shock, which involves the lungs essentially shutting down, resulting in fatality [20,21,22]. Bcc bacteria have proven to be very resilient and incredibly difficult to combat as they are resistant to almost all known antimicrobial agents and can survive under the most extreme conditions [23]. In this publication, we report a complete biodiscovery pipeline aiming at the identification of novel anti-Bcc compounds, starting from the isolation of bacteria from Antarctic sub-sea sediments. Bacteria were tested for their antimicrobial potential and a bioassay-guided purification was performed that yielded three bioactive compounds active against Bcc. Structures were then elucidated and two compounds have not been reported previously.

## 2. Results and Discussion

### 2.1. Isolation of Bacteria, Typing and Phylogenetic Analysis

Psychrophilic Antarctic bacteria were isolated from sediments on PYG minimal medium. After 15 days of incubation at 4 °C, 25 visible colonies where picked and grown in liquid PYG at 15 °C for 48 h in agitation, and glycerol stab were stored at −80 °C.

In order to check whether the 25 bacterial isolates represented either the same or different strains, an RAPD analysis was carried out using the primers 1253 (5′-GTTTCCGCCC-3′) and AP5 (5′-TCACGCTGCG-3′). The RAPD profiles obtained were then compared among them; the comparative analysis obtained with primer 1253 revealed that the 25 Antarctic isolates were split into 18 different RAPD groups (hereinafter, RAPD haplotypes), most of which were represented by just one bacterial isolate as summarized in Table 1. Two groups embedding more than one isolate were identified: group 1 (RAPD halpotype 1) including strains BTN1, BTN6, BTN 7, BTN8, BTN9 and BTN10 and group 4 (embedding isolates BTN20A, BTN20B, and BTN24). These data were completely confirmed by the RAPD analysis performed with primer AP5.

The phylogenetic affiliation of bacterial isolates was performed through the 16S rRNA genes amplification and analysis. For this purpose, the 16S rRNA genes were PCR amplified and the nucleotide sequence of the amplicons determined. Each sequence was used as a query in a BLAST search to retrieve the most similar ones. Sequences were then aligned using the program ClustalW and the alignment was used to construct the phylogenetic trees shown in Figure S1, revealing that:

(i) As expected on the basis of the sharing of RAPD profiles, the six strains exhibiting the same RAPD profile (RAPD haplotype 1) share the same 16S rRNA gene sequence and were clustered together joining the species *Pseudomonas* *azotoformans*.
(ii) Strain BTN4 was affiliated to the genus *Arthrobacter*.
(iii) All the other strains were affiliated to the genus *Psychrobacter* and, according to the different RAPD profiles they exhibited, joined different *Psychrobacter* clades. The three strains (BTN20A, BTN24 and BTN 20B) sharing the same RAPD profile (RAPD haplotype 4), joined the same *Psychrobacter* cluster.

### 2.2. Cross-Streaking Experiments

In order to check the ability of Antarctic bacteria to inhibit the growth of Bcc strains, cross-streaking experiments were performed using representatives of each RAPD haplotype as test strains. We used as targets a panel of 84 different Bcc strains belonging to 17 known species (see Table S1). Most of the strains had a clinical origin. Data obtained are summarized in Table S1, revealing that all BTN strains are able to completely inhibit the growth of Bcc strains. In order to check whether this anti-Bcc activity was due to Volatile Organic Compounds (VOCs) synthesis, a further cross-streaking experiment was performed using Petri dishes with a central septum, which physically separates the tester (Antarctic) from the target strains. To perform this analysis, we selected the 17 Bcc type strains listed in Table S2, which are highlighted in red. Data obtained are reported in Table 2 and revealed that the inhibitory power of the BTN strains decreased in the presence of the central septum. This finding suggested that BTN strains synthesize a combination of volatile and soluble molecules and that the Bcc-inhibitory activity likely might rely principally on the soluble fraction. Thus, we decided to concentrate our efforts on the soluble molecules for this study.

### 2.3. Extracts’ Antimicrobial Assays

Eight of the most active Antarctic strains belonging to the three different genera (*Pseudomonas*, *Psychrobacter*, and *Arthrobacter*) were selected and used to produce extracts, which were then tested against a reduced panel of Bcc type-strains isolated from CF patients. The MIC assays were carried out as described in Materials and Methods. Table 3 reports the antimicrobial activity as percentage of Bcc growth inhibition in the presence of each extract at a concentration of 1 mg/mL.

Data obtained revealed that the extracts were differentially active against the selected Bcc strains. Three Antarctic bacterial strains, *i.e.*, BTN2, BTN15, and BTN5, were able to inhibit at least three of the five Bcc strains more than 70% of growth. However, the extract from *Pseudomonas* BTN1 exhibited the best anti-Bcc activity; indeed, it was able to almost completely inhibit the growth of all the target strains at the concentration used. For this reason, this strain was selected for further scale-up and extract purification.

### 2.4. Bioassay-Guided Purification of BTN1 Extract

*Pseudomonas* sp. BTN1 strain was grown in 3 L TYP medium for five days at 20 °C; then, the culture broth was extracted with ethyl acetate. Subsequently, the crude extract (1 g) was fractionated with an SPE C18 Cartridge. Elution was performed stepwise with an increasing methanol concentration. The four eluted fractions were collected, dried and dissolved in DMSO to perform bioassay at a stock concentration of 50 mg/mL. The fraction eluted at 100% methanol was shown to be the most active one against *B. cenocepacia* LMG 16656 with a MIC of 50 μg/mL and was subjected to HPLC separation. HPLC chromatograms extracted from 200 to 400 nm presented 11 different peaks, which were separated, dried and dissolved in DMSO at a stock concentration of 10 mg/mL to perform MIC assay. Data obtained revealed promising inhibitory activity against *B. cenocepacia* strain LMG 16656 of three compounds, hereinafter referred to as Compound **1**, **2** and **3**, respectively.

### 2.5. Compound Structure Elucidation

**Figure 1:**
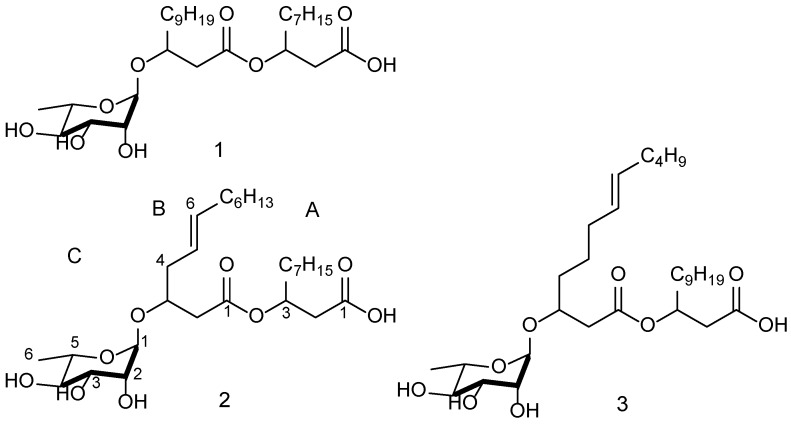
Structures of the three rhamnolipids isolated from *Pseudomonas* BTN1.

Compounds structures are shown in Figure 1. The molecular formula of compound **1** was established as C_28_H_52_O_9_ by HRESIMS (555.3514 Δ 0.92 ppm [M + Na]^+^. Dereplication of this compound based on 1D, 2D NMR and LC-MS data indicated that it is a known rhamnolipid [24] containing two fully saturated lipid chains. The chain lengths of lipids A and B of compound **1** contained 10 and 12 carbons respectively were confirmed by analysis of MS/MS fragmentation data (Figure S19). The molecular formula of compound **2** was established as C_28_H_50_O_9_ by high-resolution electrospray ionization mass spectrometry (HRESIMS) (553.3343 Δ −0.75 ppm [M + Na]^+^) and subsequent dereplication suggested it was new. The molecular formula suggested four degrees of unsaturation.

The ^1^H, ^13^C-NMR data (Table 1) in CD_3_OD of **2** revealed one ester (δ_C_ 173.4 ppm), one carboxylic acid group (δ_C_ 171.40) ppm, two olefinic carbons (δ_C_ 132.8, 123.7 ppm), and an anomeric carbon (δ_C_ 98.47 ppm) of a sugar unit. This analysis accounted for three of the double bond equivalents, suggesting that the sugar unit was present as a ring. The structure of compound **2** was elucidated based on 2D NMR correlation experiments. Data clearly showed three distinctive spin systems. There were COSY correlations observed between the anomeric proton and the adjacent protons of the sugar unit. There was a strong observed COSY correlation between the methyl group at δ_H_ 1.27 ppm and the proton at δ_H_ 3.38 ppm placing the methyl group at position C5. The proposed structure was fully supported by COSY and HMBC correlations (Table 4 and Figure S10) indicating that compound **2** is a rhamnolipid with the A and B chains having 10 and 12 carbons respectively, and a single unsaturation at position B5. Further evidence supporting the structure came from careful interpretation of MS/MS fragmentation data (Figure S21). The relative orientation of the rhamnose moiety in compound **2** was identified as α based on detailed analysis of ROE data (2D ROESY), chemical shifts and proton coupling constants [24,25]. Analysis of ROESY data showed an ROE correlation between H-1 and H-2 suggesting that both occupied equatorial positions. This is in agreement with the observed small coupling constant ^3^*J*(1,2) of about 1.4 Hz in CD_3_OD and a broad singlet in DMSO-d_6_. A ROE correlation between H-3 and H-5 suggested that both were in axial positions (Figure S13). In addition, ROE correlations were observed between H-3 and H-2 (axial-equatorial), H-6 and H-5 (equatorial-axial) and between H-6 and H-4 (equatorial-axial). This conformation is in agreement with observed coupling constants: ^3^*J*(2,3, 3.5 Hz), ^3^*J*(3,5, 9.5 Hz) and ^3^*J*(4,5, 9.8 Hz). All the data is consistent with the rhamnose unit oriented in a α position. A 2D ROESY NMR spectrum is available in the supporting information (Figure S12).

The molecular formula of compound **3** was established as C_30_H_54_O_9_ by HRESIMS 581.3649 Δ 1.72 ppm [M + Na]^+^. Based on 1D, 2D NMR and LC-MS data compound **3** was similar to **2**, the difference being an additional C_2_H_4_ unit. However, careful interpretation of the data indicated that both the lipid chains A and B were C12 carbons with a single unsaturation at position B7 instead of C10 and C12 carbons and an unsaturation position at B5 in **2**. Further evidence of this structure came from MS/MS fragmentation data (Figure S23). The relative configuration of the rhamnose unit was similar to that of compounds **1** and **2** based on similarities of chemical shifts and proton coupling constants. In all three compounds, it is assumed that the rhamnose moieties have the normal l-configuration. In addition, the absolute stereochemistry of position H-3 in the lipid chains A and B have not been determined.

### 2.6. Antimicrobial Activity of BTN1 Pure Compounds

The three monorhamnolipids isolated from strain BTN1 were tested against a selected panel of Bcc strains isolated from CF patients and *S. aureus*. MIC and MBC values are reported in Table 5. It is worth noticing that the three compounds have identical MIC and MBC values indicating a bactericidal effect against the target bacteria, as reported for several natural biosurfactants [26,27]. Compounds **2** and **1** were the most active compounds as they were effective against all the tested stains, with the only exception of *B. diffusa*. Specifically, compounds **2** and **1** had the lowest MBC values against *B. cenocepacia* (3.12 μg/mL) and *S. aureus* (respectively, 3.12 and 1.56 μg/mL). Compound **3** had antimicrobial effect only against *S. aureus* with an MBC value of 100 μg/mL, while it resulted in being ineffective towards Bcc strains. Rhamnolipids (RLs) are well-known secondary metabolites synthesized by members of different Gram-negative species, particularly from bacteria belonging to the genus *Pseuedomonas*. They perform several potential functions in bacteria: as powerful biosurfactants, they are involved in the uptake and b tested stains, with the only exception of polymerase and 0.6 iodegradation of poorly soluble substrates and are essential for surface motility and biofilm development [28]. Recently, they have emerged as potential antimicrobials against a broad range of pathogens such as *Staphylococcus*, *Mycobacterium*, and *Bacillus*, and significant activity against a number of Gram-negative species, including *Serratia marcescens*, *Enterobacter aerogenes*, and *Klebsiella pneumoniae* [29,30,31]. RLs act like synthetic surfactants and their proposed mechanism of action consists of intercalation into biological membranes and destruction by their permeabilizing effect leading to cell death [32].

## 3. Experimental Section

### 3.1. Isolation of Bacterial Strains

The Antarctic bacterial strains used in this study were isolated from environmental samples collected at −20 m of depth (sub-sea sediments) near the Mario Zucchelli Station, Baia Terranova, Ross sea, Antarctica (74.6936° S, 164.1117° E). 1 gr of sediments was mixed with 20 mL of M9 salts solution (KH_2_PO_4_ 3.0 g/L, Na_2_HPO_4_6.0 g/L, NaCl 0.5 g/L, NH_4_Cl 1.0 g/L) in a 50 mL Falcon tube and gently mixed; the supernatant was serially diluted in sterile M9 buffer and plated on PYG medium (Peptone 5.0 g/L, Yeast extract 4.0 g/L, Glucose 1.0 g/L, CaCl_2_ 0.2 g/L, MgSO_4_.7H_2_O 0.4 g/L, K_2_HPO_4_ 1.0 g/L, KH_2_PO_4_ 1.0 g/L, NaHCO_3_10.0 g/L NaCl 2.0 g/L and 17 g/L). After 15 days of incubation, 24 visible colonies were picked, grown in liquid PYG and stored at −80 °C.

### 3.2. Target Strains and Growth Conditions

Bcc strains used in this work are listed in Table 2 and Table S1. Bcc and *S. aureus* 6538P were routinely grown on Luria-Bertani broth (LB) (Tryptone 10 g/L, Yeast extract 5 g/L, NaCl 10 g/L) at 37 °C. BTN isolated Antactic strains were routinely grown in TYP medium (Bacto-tryptone 16 g/L, 16 g/L Yeast extract, 10 g/L NaCl) and Marine Broth (MB) at 21 °C. To allow bacterial growth on solid media, 17 g/L of bacteriological agar were added to each medium.

### 3.3. RAPD Analysis

Typing of bacterial isolates was performed using the Random Amplified Polymorphic DNA (RAPD) technique performed on cell lysates [33,34,35]; to this purpose, Antarctic bacterial colonies grown overnight at 21 °C on MA plates were suspended in 25 μL of sterile distilled water, heated to 95 °C for 10 min, and cooled on ice for 5 min. RAPD analysis was carried out in a total volume of 25 μL containing 1× Reaction Buffer, 300 μM MgCl_2_, 200 μM of each deoxynucleoside triphosphate, 0.5 U of Polytaq DNA polymerase (Polymed, Florence, Italy), 10 μM of primer 1253 (5′GTTTCCGCCC3′) or primer AP5 (5′TCACGCTGCG3′) and 2 μL of lysate cell suspension [35]. PCR were performed using MasterCycle Personal Thermal Cycler (Eppendorf, Hamburg, Germany). After incubation at 90 °C for 1 min and 95 °C for 1.5 min, the reaction mixtures were cycled 45 times through the following temperature profile: 95 °C for 30 s, 36 °C for 1 min, and 75 °C for 1 min. Samples were then incubated at 60 °C for 10 min, and finally at 5 °C for 10 min. Amplification products were then stored at −20 °C. Reaction products were analyzed by agarose (2.5 % *w*/*v*) gel electrophoresis in TAE buffer containing 0.5 μg/mL (*w*/*v*) of ethidium bromide.

### 3.4 Phylogenetic Affiliation of BTN Strains

Two μL of each cell lysate were used for the amplification via PCR of 16S rRNA genes. PCR was carried out in a total volume of 50 μL containing 1X Reaction Buffer, 150 μM MgCl_2_, 250 μM of each deoxynucleoside triphosphate, and 2.0 U of Polytaq DNA polymerase and 0.6 μM of primer P0 (5′GAGAGTTTGATCCTGGCTCAG3′) and P6 (5′CTACGGCTACCTTGTTACGA3′) [36]. The reaction conditions used were: one cycle (95 °C for 90 s), 30 cycles (95 °C 30 s, 50 °C 30 s, and 72 °C 1 min), with a final extension of 10 min at 72 °C. Amplicons corresponding to the 16S rRNA genes (observed under UV light, 312 nm) were excised from the gel and purified using the “QIAquick” gel extraction kit (QIAGEN, Chatsworth, CA, USA) according to manufacturer’s instructions. Direct sequencing was performed on both DNA strands using an ABI PRISM 310 Genetic Analyzer (Applied Biosystems, Forster City, CA, USA) and the chemical dye terminator [37]. Each 16S rRNA gene sequence was submitted to GenBank and assigned the accession number shown in Table 1. BLAST probing of DNA databases was performed with the BLASTn option of the BLAST program using default parameters [38]. Nucleotide sequences were retrieved from RDP databases. The ClustalW program was used to align the 16S rRNA gene sequences obtained with the most similar ones retrieved from the databases [39]. Each alignment was checked manually, corrected, and then analyzed. The evolutionary history was inferred using the Neighbor-Joining method according to the model of Kimura two-parameter distances [40,41]. The percentage of replicate trees where the associated taxa clustered together in the bootstrap test (1000 replicates) is shown next to the branches [42].

### 3.5. Cross-Streaking

Cross-streaking experiments were carried out as previously described [11]. Petri dishes with or without a septum separating two hemi-cycles were used. Plates with a central septum allowed the growth of tester and target strains without any physical contact. Antarctic strains (tester strains) were grown on MA for four days at 21 °C; then, they were streaked on TYP and incubated at 21 °C for four days. Bcc strains (target strains) were perpendicularly streaked to the initial streak and plates were further incubated at 21 °C for two days and at 37 °C for two additional days. The experiments were conducted in parallel with a positive control to verify the viability of Bcc cells.

### 3.6. Extract Preparation

A single colony of a bacterial isolate was used to inoculate 3 mL of liquid TYP media in sterile bacteriological tubes. After 48 h of incubation at 21 °C at 200 rpm, the pre-inoculum was used to inoculate 100 mL of TYP medium in a 500 mL flask, at an initial cell concentration of 0.01 OD_600_/mL. The flasks were incubated up to five days at 21 °C at 200 rpm. The cultures were then centrifuged at 6800× *g* at 4 °C for 30’, and the exhausted culture broths were collected and stored at −20 °C. The exhausted culture broths were subjected to organic extraction using three volumes of ethyl acetate in a 500 mL separatory funnel. The organic phase collected was evaporated using a Laborota 4000 rotary evaporator (Heidolph, Schwabach, Germany), and the extracts were weight, dissolved in 100% DMSO at 50 or 100 mg/mL and stored at −20 °C.

### 3.7. Antimicrobial Assays

#### 3.7.1. Minimal Inhibitory Concentration Assay (MIC)

To evaluate the antimicrobial potential of Antarctic extracts, samples were placed into each well of a 96-well microtiter plate at an initial concentration of 2% (*v*/*v*) and serially diluted using LB medium. Wells containing no compound represented the negative control. DMSO was used as control to determine the effect of solvent on cell growth. A single colony of a Bcc strain was used to inoculate 3 mL of liquid LB media in sterile bacteriological tube. After 6–8 h of incubation, growth was measured by monitoring the absorbance at 600 nm and about 40,000 CFU were dispensed in each well of the prepared plate. Plates were incubated at 37 °C for 24 h and growth was measured with a Cytation3 Plate Reader (Biotek, Winoosky, VT, USA) by monitoring the absorbance at 600 nm.

#### 3.7.2. Minimal Bactericidal Concentration (MBC) Assay

To determine the MBC, the dilution representing the MIC and two of the more concentrated test product dilutions were plated on LB agar plates and enumerated to determine CFU/mL. An aliquot of the positive control was plated and used to establish a baseline concentration of the microorganism used.

### 3.8. Purification of Ethyl-Acetate Crude Extract

Crude extract of 3 L BTN1 fermentation, prepared as described above, was subjected to fractionation using Chromabond SPE C18 column cartridges (Macherey-Nagel, Duren, Germany). HPLC separations were carried out using a VP 250/10 Nucleodur C18 HTec, 5 μm, (Macherey-Nagel Duren, Germany) connected to a Ultimate 3000 HPLC Chromatograph with a Ultimate 3000 Diode Array detector and in-line degasser (Dionex, Sunnyvale, CA, USA). Detection was achieved on-line through a scan of wavelengths from 200 to 400 nm. This process yielded 4.8 mg of **1**, 5.3 mg of **2** and 5.7 mg of **3**.

**Compound** **2.** [α]_D_ −53.4° (*c* 0.001 MeOH; UV(MeOH) λ_max_ (log ε) 202 (3.55) nm; IR (film) υmax 3361, 2925, 2855, 1735, 1671, 1575, 1455, 1380, 1314, 1207, 1161, 1126, 1037, 983, cm^−1^; ^1^H, ^13^C, HMBC NMR data see Table 1; HRESIMS *m*/*z* 553.3343 Δ −0.75 ppm [M + Na]^+^ calculated for C_28_H_50_O_9_.

**Compound** **3.** [α]_D_ +49.3° (*c* 0.001 MeOH UV(MeOH) λ_max_ (log ε) 202 (3.78) nm; IR (film) υmax 3387, 2926, 2855, 1667, 1587, 1402, 1316, 1204, 1130, 1072, 1049, 983 cm^−1^; ^1^H, ^13^C, HMBC NMR data see Table 1; HRESIMS *m*/*z* 581.3649 Δ 1.72 ppm [M + Na]^+^ calculated for C_30_H_54_O_9_.

### 3.9. NMR–LCMS Experiments

NMR data, both 1D and 2D were recorded on a spectrometer (Bruker, Billerica, MA, USA) at 600 and 150 MHz for ^1^H and ^13^C, respectively, using an ID cryoprobe in methanol-d_4_ as solvent. Chemical shifts are reported in parts per million (δ/ppm) downfield relative to residual CD_3_OD at 3.31 ppm for protons and 49.0 ppm for carbons. High-resolution mass spectrometry and fragmentation data were recorded using an LTQ Orbitrap system (ThermoScientific, Whaltman, MA, USA) coupled to an 1290 Infinity HPLC system (Agilent, Santa Clara, CA, USA). The following conditions were used: capillary voltage 45 V, capillary temperature 320 °C, auxiliary gas flow rate 10–20 arbitrary units, sheath gas flow rate 40–50 arbitrary units, spray voltage 4.5 kV, mass range 100–2000 amu (maximum resolution 30,000). Optical rotation measurements were recorded using a Perkin Elmer, Model 343 Polarimeter at 589 nm (Perkin Elmer, Whaltman, MA, USA). The UV spectrum was recorded on a UV-Vis spectrophotometer model S10 (Spectromlab, Barcelona, Spain). The IR was recorded on a PerkinElmer FTIR Spectrum Two instrument (Perkin Elmer, Whaltman, MA, USA).

## 4. Conclusions

Exploiting a bioassay-driven purification approach, three RLs (one of which was novel) with antimicrobial activity against Bcc strains, were isolated from *Pseudomonas* sp. BTN1, recovered from Antarctic sediments. RLs represent a promising class of biosurfactants as antimicrobials or in combination with antibiotics. A recent study suggested the use of RLs as an additive in the formulation of antibiotic and other antimicrobial agents for enhancing the effectiveness of chemotherapeutics [43]. Moreover, the possibility of RL production by the fermentation of organic waste (such us waste oils), makes these products economically appealing [44]. To the best of our knowledge, this is the first report of antimicrobial activity of RLs against Bcc strains, and it prompts future studies aimed at RL exploitation as drugs to counteract these hazardous opportunistic human pathogens.

## Figures and Tables

**Table 1:** List of the strains used in this work; for each strain, the genus and the RAPD haplotype are reported.

Genus	Strains	RAPD Profile	Accession Number
*Pseudomonas*	BTN1	1	KT989002
	BTN6		KT989003
	BTN7		KT989004
	BTN8		KT989005
	BTN9		KT989006
	BTN10		KT989007
*Psychrobacter*	BTN3	2	KT989009
	BTN19	3	KT989019
	BTN20B	4	KT989021
	BTN24		KT989022
	BTN21	5	KT989025
	BTN23	6	KT989024
	BTN2	7	KT989008
	BTN11	8	KT989011
	BTN5	9	KT989010
	BTN20A	4	KT989020
	BTN15	10	KT989015
	BTN13	11	KT989012
	BTN14	12	KT989013
	BTN17	13	KT989017
	BTN16	14	KT989016
	BTN18	15	KT989018
	BTN12	16	KT989014
	BTN22	17	KT989023
*Arthrobacter*	BTN4	18	KT989001

**Table 2:** Growth of Bcc strains in cross-streaking experiments carried out using Petri dishes either with (W) or without (N) a central septum (S). Symbols: +, growth; ±, reduced growth; -, no growth.

Bcc Strain	S	BTN Strain																			
		1	2	3	5	11	13	14	4	12	15	16	17	18	19	20 a	20 b	21	22	23	C+
*B. ambifaria* LMG 19182	W	-	-	-	-	-	-	-	-	-	-	-	-	-	-	-	-	-	-	-	+
	N	-	-	-	-	-	-	-	-	-	-	-	-	-	-	-	-	-	-	-	+
*B. anthina* LMG 20980	W	-	-	-	-	-	-	-	-	-	-	-	-	-	-	-	-	-	-	-	+
	N	-	-	-	-	-	-	-	-	-	-	-	-	-	-	-	-	-	-	-	+
*B. vietnamensis* LMG10929	W	-	-	-	-	-	-	-	-	-	-	-	-	-	-	-	±	-	-	-	+
	N	-	-	-	-	-	-	-	-	-	-	-	-	-	-	-	-	-	-	-	+
*B. cenocepacia* LMG 16656	W	±	-	±	±	-	±	-	-	-	-	-	±	±	-	±	±	-	-	-	+
	N	-	-	-	-	-	-	-	-	-	-	-	-	-	-	-	-	-	-	-	+
*B. cepacia* LMG 1222	W	±	±	±	±	±	±	±	±	±	±	±	±	±	±	±	±	±	±	±	+
	N	-	-	-	-	-	-	-	-	-	-	-	-	-	-	-	-	-	-	-	+
*B. contaminas* LMG 23361	W	±	±	±	±	±	±	±	±	±	±	±	±	±	±	±	±	±	±	±	+
	N	-	-	-	-	-	-	-	-	-	-	-	-	-	-	-	-	-	-	-	+
*B. diffusa* LMG 24065	W	±	±	±	±	±	±	±	±	±	±	±	±	±	±	±	±	±	±	±	+
	N	-	-	-	-	-	-	-	-	-	-	-	-	-	-	-	-	-	-	-	+
*B. dolosa* LMG 18943	W	±	±	±	±	±	±	±	±	±	±	±	±	±	±	±	±	±	±	±	+
	N	-	-	-	-	-	-	-	-	-	-	-	-	-	-	-	-	-	-	-	+
*B. lata *LMG 22485	W	±	±	±	±	±	±	±	-	±	±	±	±	±	±	±	±	±	±	±	+
	N	-	-	-	-	-	-	-	-	-	-	-	-	-	-	-	-	-	-	-	+
*B. latens* LMG 24064	W	-	-	±	±	±	-	-	-	-	±	±	±	±	-	±	±	-	-	±	+
	N	-	-	-	-	-	-	-	-	-	-	-	-	-	-	-	-	-	-	-	+
*B. metallica* LMG 24068	W	±	±	±	±	±	±	±	±	±	±	±	±	±	±	±	±	±	±	±	+
	N	-	-	-	-	-	-	-	-	-	-	-	-	-	-	-	-	-	-	-	+
*B. multivorans* LMG 13010	W	-	±	±	±	±	±	±	-	±	±	±	±	±	±	±	±	±	±	±	+
	N	-	-	-	-	-	-	-	-	-	-	-	-	-	-	-	-	-	-	-	+
*B. pseudomultivorans* LMG 26883	W	±	±	±	±	±	±	±	±	±	±	±	±	±	±	±	±	±	±	±	+
	N	-	-	-	-	-	-	-	-	-	-	-	-	-	-	-	-	-	-	-	+
*B. pyrrocinia* LMG 14191	W	±	±	±	±	±	±	±	±	±	±	±	±	±	±	±	±	±	±	±	+
	N	-	-	-	-	-	-	-	-	-	-	-	-	-	-	-	-	-	-	-	+
*B. seminalis* LMG 24067	W	-	±	±	±	±	±	±	-	±	-	±	±	±	±	±	-	±	±	±	+
	N	-	-	-	-	-	-	-	-	-	-	-	-	-	-	-	-	-	-	-	+
*B. stabilis* LMG 14294	W	±	±	±	±	±	±	±	±	±	±	±	±	±	±	±	-	±	±	±	+
	N	-	-	-	-	-	-	-	-	-	-	-	-	-	-	-	-	-	-	-	+
*B. uborrensis* LMG 20358	W	-	-	±	±	±	±	±	-	±	±	±	±	±	-	±	±	±	±	±	+
	N	-	-	-	-	-	-	-	-	-	-	-	-	-	-	-	-	-	-	-	+

**Table 3:** Antimicrobial activity of BTN cell extracts reported as % of inhibition of Bcc strains treated with 1 mg/mL of BTN extracts.

		*Pseudomonas*	*Psychrobacter*						*Arthrobacter*
Species	Strain	BTN 1	BTN 2	BTN 15	BTN 3	BTN 19	BTN 21	BTN 5	BTN 4
*B. diffusa*	LMG 24065	100 ± 0	75 ± 3	77 ± 3	43 ± 7	45 ± 11	70 ± 4	77 ± 9	63 ± 3
*B. metallica*	LMG 24068	92 ± 4	70 ± 5	71 ± 3	32 ± 2	30 ± 3	53 ± 5	77 ± 4	64 ± 9
*B. cenocepacia*	LMG 16656	100 ± 0	78 ± 2	87 ± 1	84 ± 6	64 ± 4	45 ± 1	84 ± 2	57 ± 1
*B. latens*	LMG 24064	100 ± 0	53 ± 11	75 ± 2	55 ± 6	43 ± 3	65 ± 2	56 ± 3	41 ± 2
*B. seminalis*	LMG 24067	100 ± 0	43 ± 6	67 ± 5	73 ± 8	45 ± 6	78 ± 11	40 ± 3	56 ± 3

**Table 4:** NMR data of **2** and **3** in CD_3_OD. ^a^ 150 MHz; ^b^ 600 MHz.

		2				3			
	Position	™_C_/ppm ^a^, m	™_H/_ppm (m, *J* in Hz) ^b^	COSY ^1^H–^1^H	HMBC H→C	™_C_/ppm ^a^, m	™_H/_ppm (m, *J* in Hz) ^b^	COSY ^1^H–^1^H	HMBC H→C
A	1	173.4, C				175.5, C			
	2	38.9, CH_2_	2.58, m	A3	A1	40.9, CH_2_	2.54, m	A3	A1
	3	71.1, CH	5.27, pentet, 6.4	A2, A3	A1, A2	72.7, CH	5.29, pentet, 6.5	A2, A4	A1, A2
	4	33.8, CH_2_	1.64, m	A3	A3	34.9, CH_2_	1.63, bm	A3	A3
	5	24.9, CH_2_	1.35, overlap			26.0, CH_2_	1.35, overlap		
	6	29.3, CH_2_	1.31, overlap			30.5, CH_2_	1.37, overlap		
	7	29.3, CH_2_	1.31, overlap			30.1, CH_2_	1.32, overlap		
	8	31. 6, CH_2_	1.31, overlap			29.8, CH_2_	1.33, overlap		
	9	22.3, CH_2_	1.33, overlap	A10	A10	30.2, CH_2_	1.36, overlap	A10	A10
	10	13.1, CH_3_	0.92, m	A9	A9	32.7, CH_2_	1.31, overlap	A9	A9
	11					23.4, CH_2_	1.33, overlap		
	12					14.1, CH_3_	0.92, m		
B	1	171.4, C				172.3, CH			
	2	39.5, CH_2_	2.53, m	B3	B1	41.0, CH_2_	A: 2.60, m B: 2.50, m	B3	B1
	3	72.9, CH	4.16, pentet, 5.8	B2, B4	B1, B5	74.8, CH	4.10, pentet, 5.9	B2, B4	B1, B5
	4	30.4, CH_2_	A: 2.39, m B: 2.33, m	B3, B5	B3, B5	33.5, CH_2_	1.58, bm	B3,B5	B3, B5
	5	123.7, CH	5.40, m	B4, B6	B3, B4, B6, B7	25.7, CH_2_	1.43, overlap	B4, B6	
	6	132.8, CH	5.55, m	B5, B7	B5, B8	27.8, CH_2_	2.08, overlap	B5, B7	
	7	27.1, CH_2_	2.08, m	B6	B5, B6	130.0, CH	5.37, m	B6, B8	B8, B6, B9
	8	29.3, CH_2_	1.31, overlap			131.2, CH	5.39, m	B7	B7
	9	28.9, CH_2_	1.33, overlap	B7		32. 7, CH_2_	1.31, overlap	B8	
	10	31.6, CH_2_	1.31, overlap			32.7, CH_2_	1.31, overlap		
	11	22.3, CH_2_	1.33, overlap		B12	23.4, CH_2_	1.33, overlap	B12	
	12	13.1, CH_3_	0.92, m		B11	14.1, CH_3_	0.92, m	B11	
C	1	98.5, CH	4.86, overlap	C2	B3, C2	100.0, CH	4.80, d, 1.4	C2	B3, C2
	2	71.2, CH	3.77, dd, 3.5, 1.4	C1, C3	C3, C4	72.4, CH	3.76, dd, 3.4, 1.4	C1, C3	C3, C4
	3	70.9, CH	3.64, dd, 9.5, 3.5	C2, C4	C5	71.9, CH	3.66, dd, 9.7, 3.4	C2, C4	C5
	4	72.7, CH	3.38, dd, 9.5, 9.8	C3,C5	C3	73.8, CH	3.35, dd, 9.7, 9.8	C3, C5	C3
	5	68.7, CH	3.67, m	C4, C6	C4, C6	69.8, CH	3.68, m	C4, C6	C4, C6
	6	16.6, CH_3_	1.27, d, 6.2	C5	C5	17.6, CH_3_	1.27, d, 6.3	C5	C5

**Table 5:** MIC and MBC values of the 3 mono-rhamnolipids isolated in this study.

Antimicrobial Activity (μg/mL)												
	*B. cenocepacia* LMG 16656		*B. metallica* LMG 24068		*B. seminalis* LMG 24067		*B. diffusa* LMG 24065		*B. latens* LMG 24064		*S. aureus* 6538P	
	MIC	MBC	MIC	MBC	MIC	MBC	MIC	MBC	MIC	MBC	MIC	MBC
C1	3.12	3.12	50	50	12.5	12.5	>200	>200	12.5	12.5	1.56	1.56
C2	3.12	3.12	25	25	3.12	3.12	200	200	12.5	12.5	3.12	3.12
C3	200	200	>200	>200	>200	>200	>200	>200	>200	>200	100	100

## Supplementary Files

Supplementary File 1
