# Supplementary material for: Antimicrobial Activity of Monoramnholipids Produced by Bacterial Strains Isolated from the Ross Sea (Antarctica)
Source: Mar Drugs. 2016 Apr 26;14(5):83. doi: 10.3390/md14050083 (PMC4882557; doi:10.3390/md14050083)
Supplement: Supplementary File 1 [file marinedrugs-14-00083-s001.pdf]

Supplementary Materials: Antimicrobial Activity of Monoramnholipids Produced by Bacterial Strains Isolated from Ross Sea (Antarctica)

Pietro Tedesco, Isabel Maida, Fortunato Palma Esposito, Emiliana Tortorella, Karolina Subko, Chidinma Christiana Ezeofor, Ying Zhang, Jioji Tabudravu, Marcel Jaspars, Renato Fani and Donatella de Pascale

Table of contents:

Figure S1. Phylogenetic trees (using the complete deletion option) of the *Arthrobacter* (A), *Pseudomonas* (B) and *Psychrobater* (C) genera.

Figure S2. ^{1}H-NMR spectrum of compound **1** in CD_3OD at 600 MHz.

Figure S3. Edited-HSQC NMR spectrum of compound **1** in CD_3OD.

Figure S4. COSY NMR spectrum of compound **1** in CD_3OD.

Figure S5. HMBC spectrum of compound **1** in CD_3OD at 600 MHz.

Figure S6. ^{1}H-NMR spectrum of compound **1** in DMSO-d6 at 400 MHz.

Figure S7. ^{1}H-NMR spectrum of compound **2** in CD_3OD at 600 MHz.

Figure S8. Edited-HSQC NMR spectrum of compound **2** in CD_3OD.

Figure S9. COSY NMR spectrum of compound **2** in CD_3OD.

Figure S10. HMBC NMR spectrum of compound **2** in CD_3OD.

Figure S11. ^{1}H-NMR spectrum of compound **2** in DMSO-d6 at 400 MHz.

Figure S12. 2D ROESY spectrum of compound **2** in DMSO-d6 with Rhamnose protons labelled.

Figure S13. Compound **2** showing selected ROE correlations of the rhamnose moiety.

Figure S14. ^{1}H-NMR spectrum of compound **3** in CD_3OD at 600 MHz.

Figure S15. Edited-HSQC spectrum of compound **3** in CD_3OD at 600 MHz.

Figure S16. COSY NMR spectrum of compound **3** in CD_3OD at 600 MHz.

Figure S17. HMBC NMR spectrum of compound **3** in CD_3OD.

Figure S18. HRESIMS spectrum $(M + Na)^+$ of compound **1**.

Figure S19. MS/MS data of compound **1**.

Figure S20. HRESIMS spectrum $(M + Na)^+$ of compound **2**.

Figure S21. MS/MS data of compound **2**.

Figure S22. HRESIMS spectrum $(M + Na)^+$ of compound **3**.

Figure S23. MS/MS data for compound **3**.

Table S1. Cross- streaking experiments of BTN isolates against a wide panel of Bcc strains. The Bcc strains highlighted in red correspond to type strains. Symbols: +, growth; ±, reduced growth; -, no growth; C+, positive controls, *i.e.*, Bcc strains grown in the absence of the tester strain(s). Abbreviations: *Ps*, *Pseudomonas*; *Ar*, *Arthrobacter*; CF, Cystic Fibrosis; Env; environmental.

Supplementary material

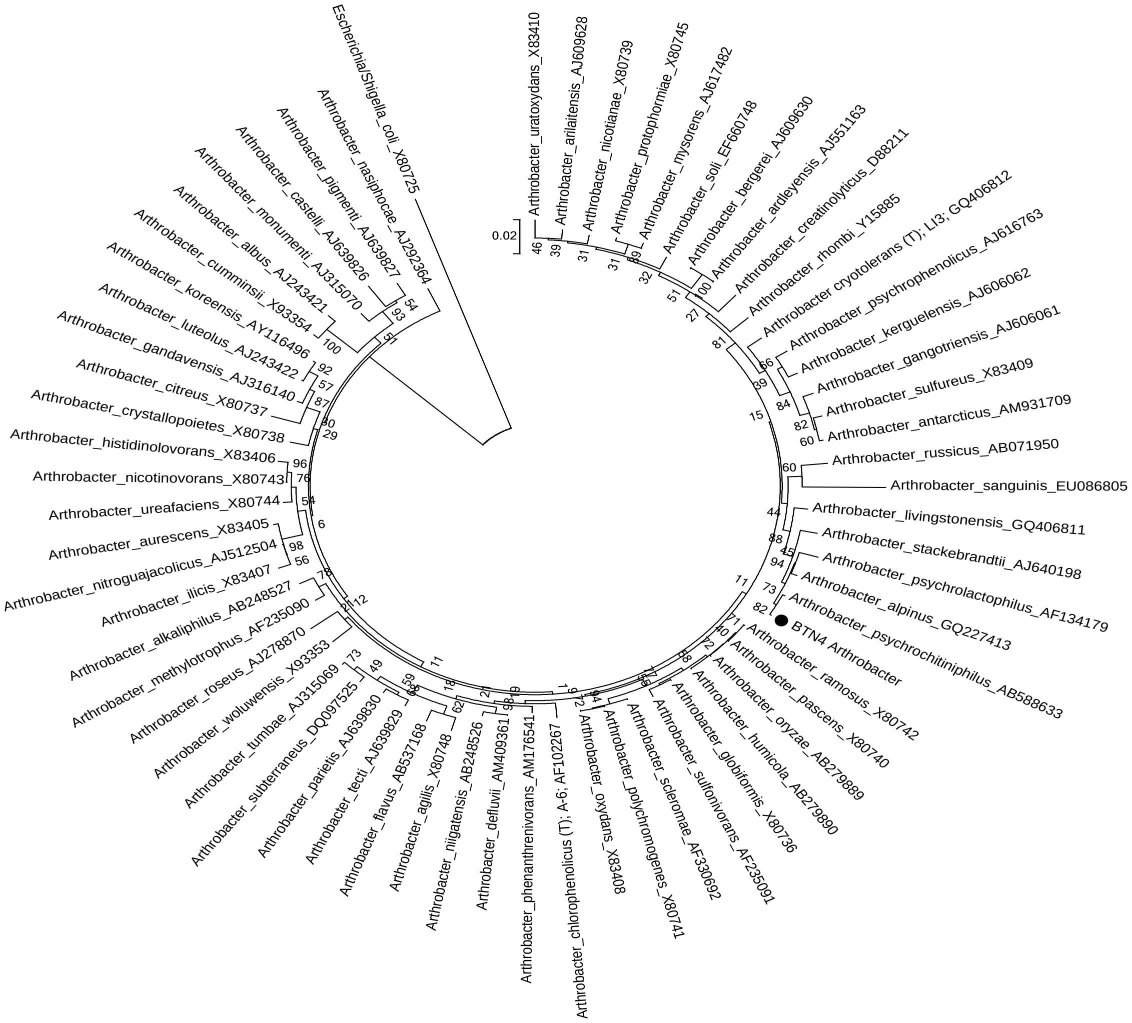

(**A**)

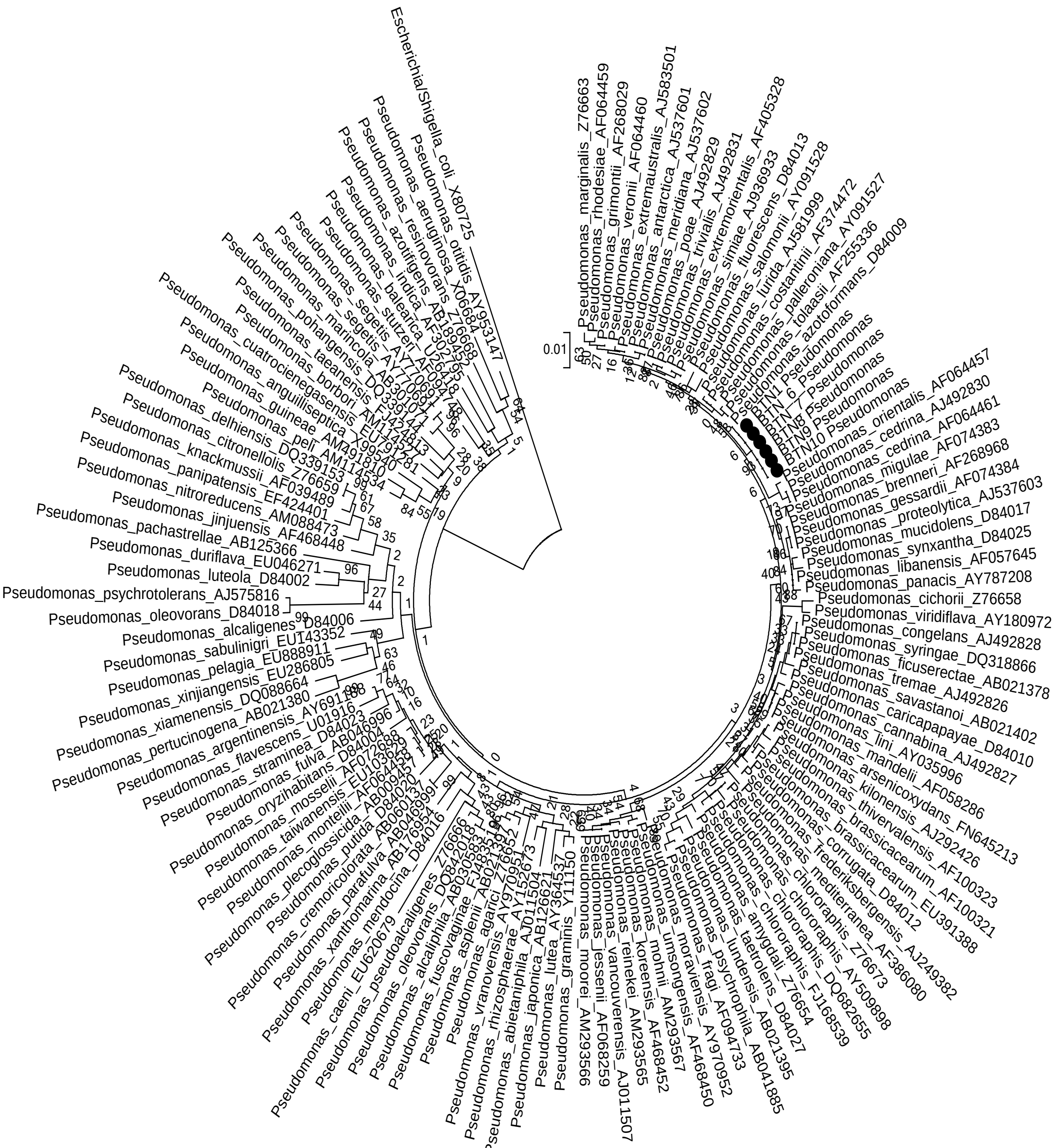

(**B**)

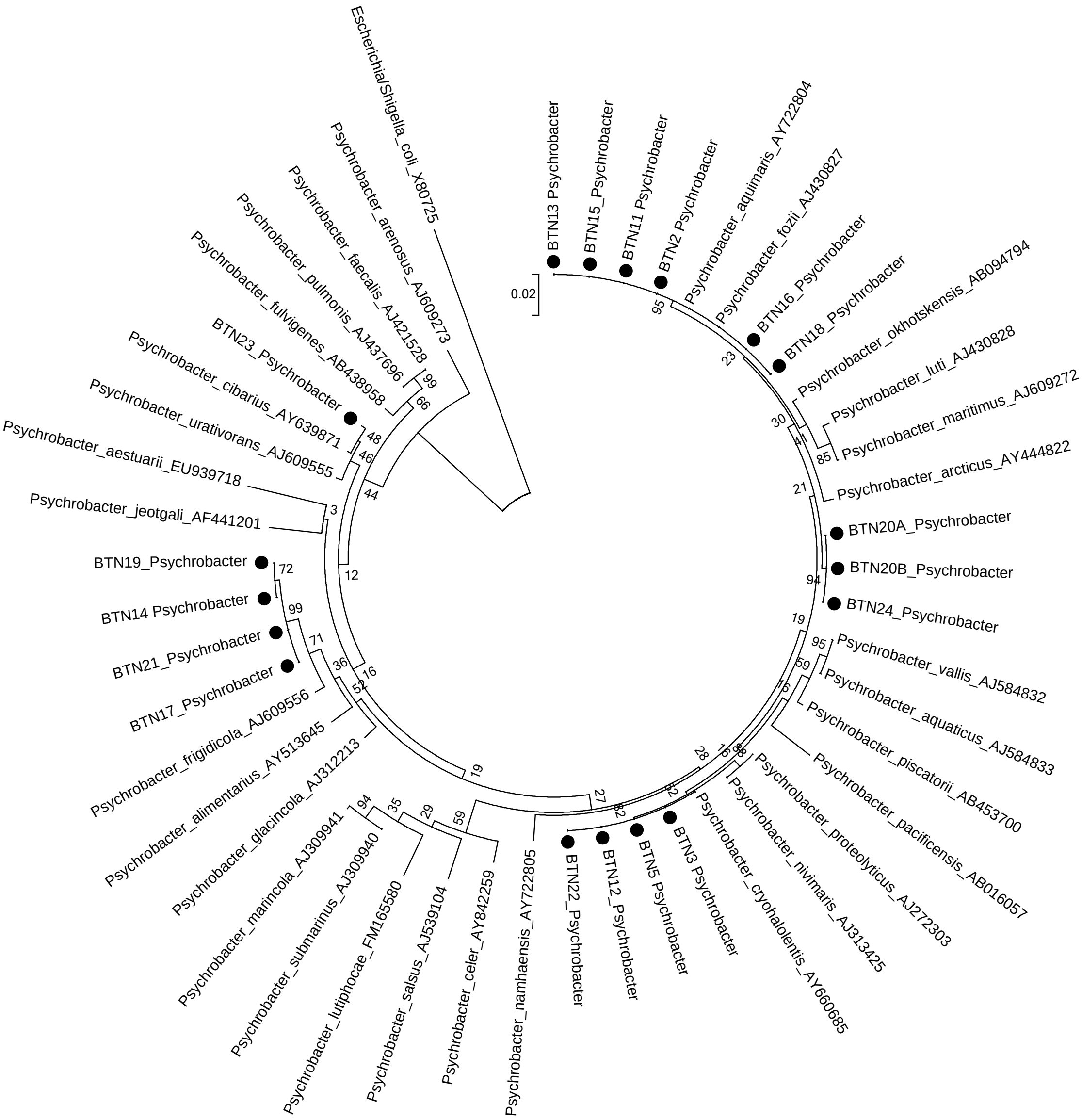

(**C**)

Figure S1. Phylogenetic trees (using the complete deletion option) of the *Arthrobacter* (**A**); *Pseudomonas* (**B**) and *Psychrobater* (**C**) genera.

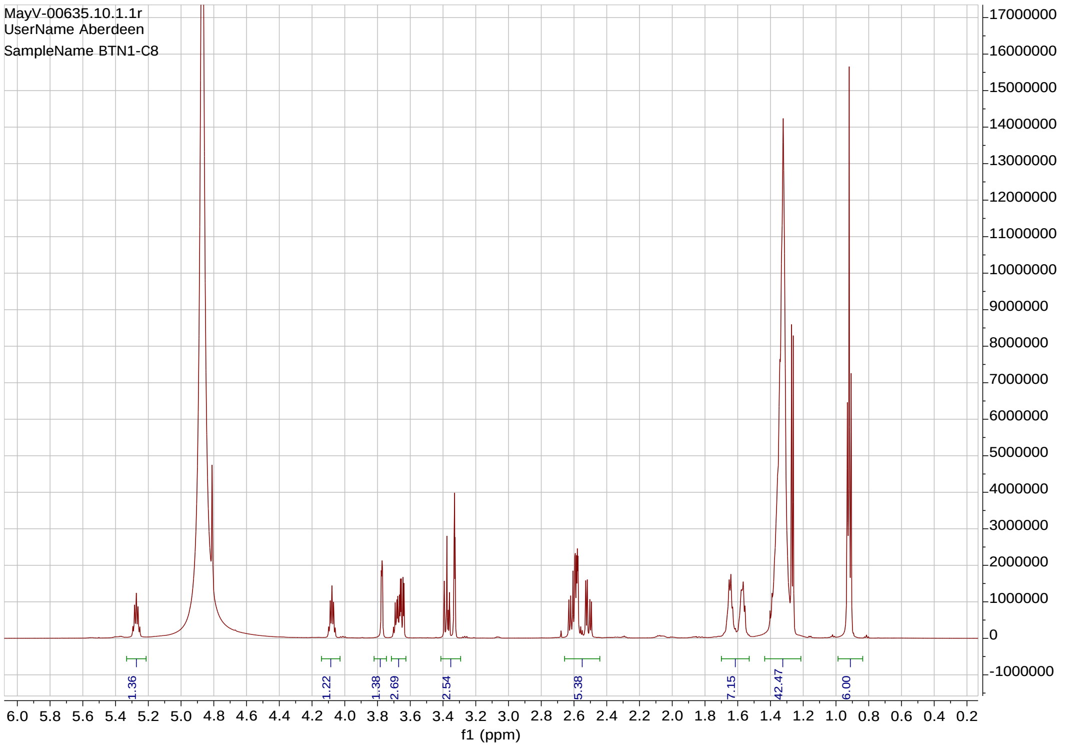

Figure S2. ^{1}H-NMR spectrum of compound **1** in CD_3OD at 600 MHz.

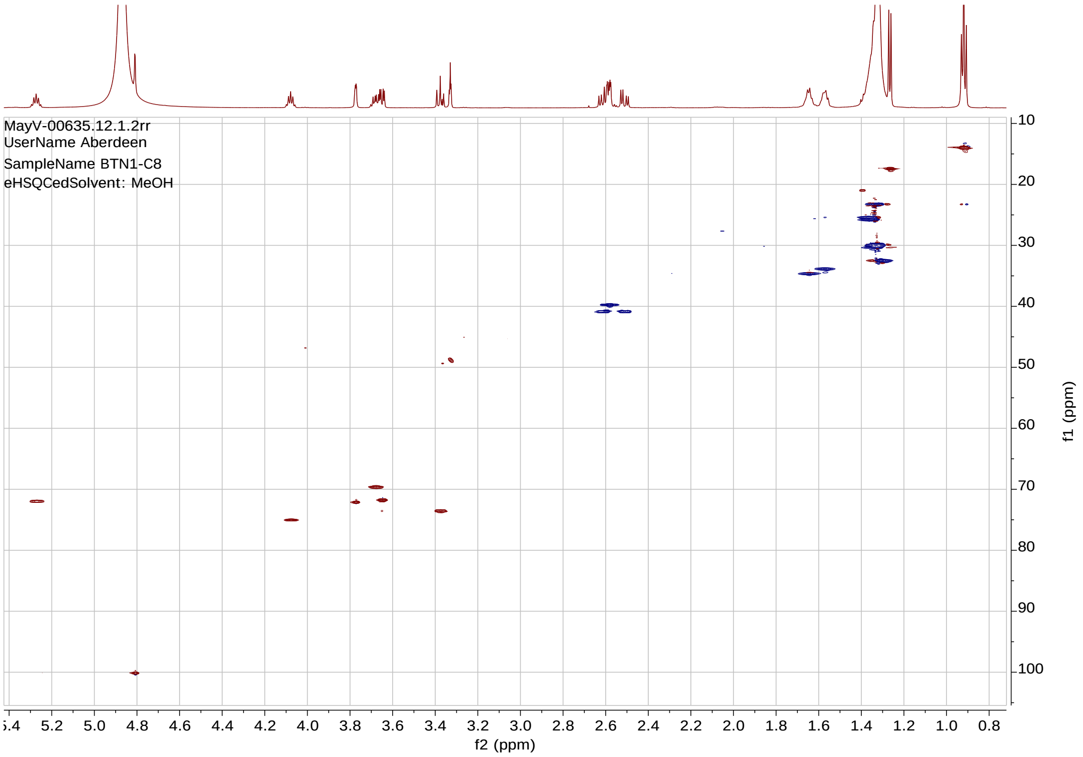

Figure S3. Edited-HSQC spectrum of compound **1** in CD_3OD at 600 MHz.

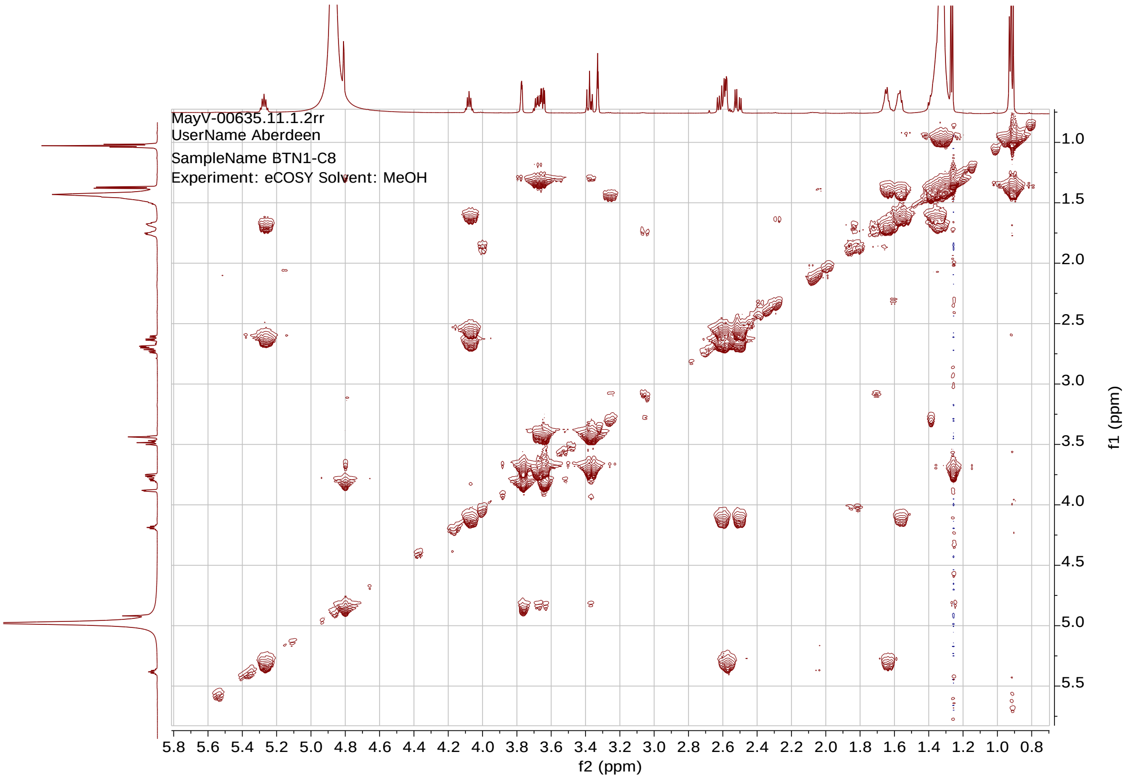

Figure S4. COSY NMR spectrum of compound **1** in CD_3OD at 600 MHz.

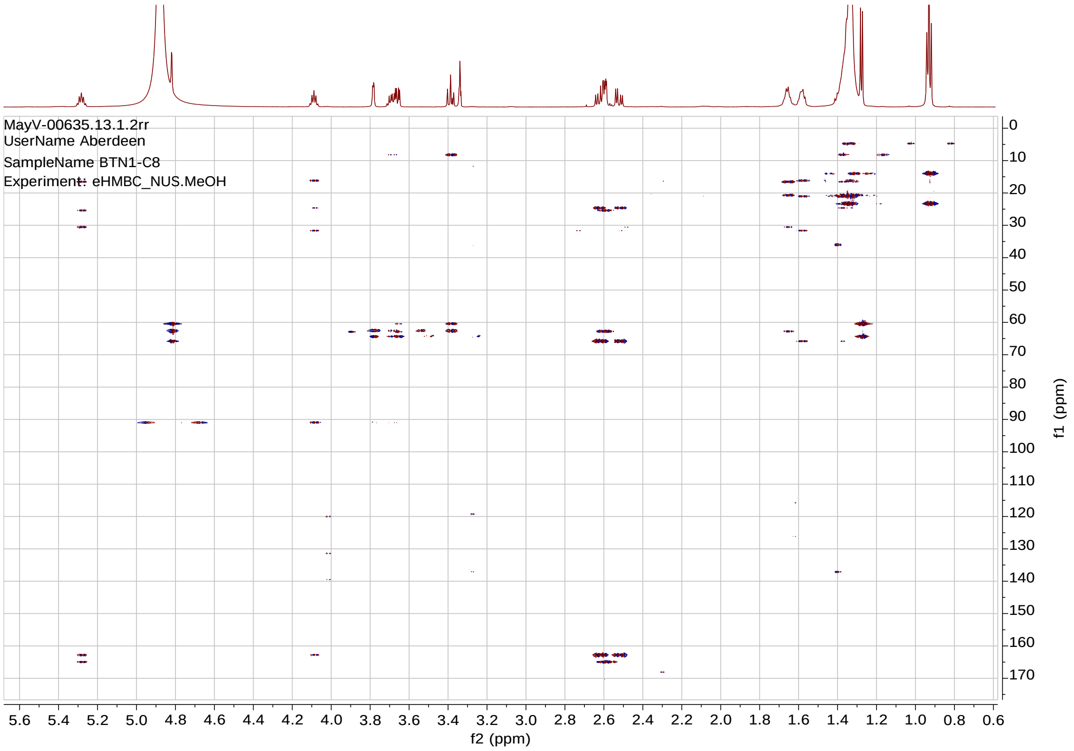

Figure S5. HMBC spectrum of compound **1** in CD_3OD at 600 MHz.

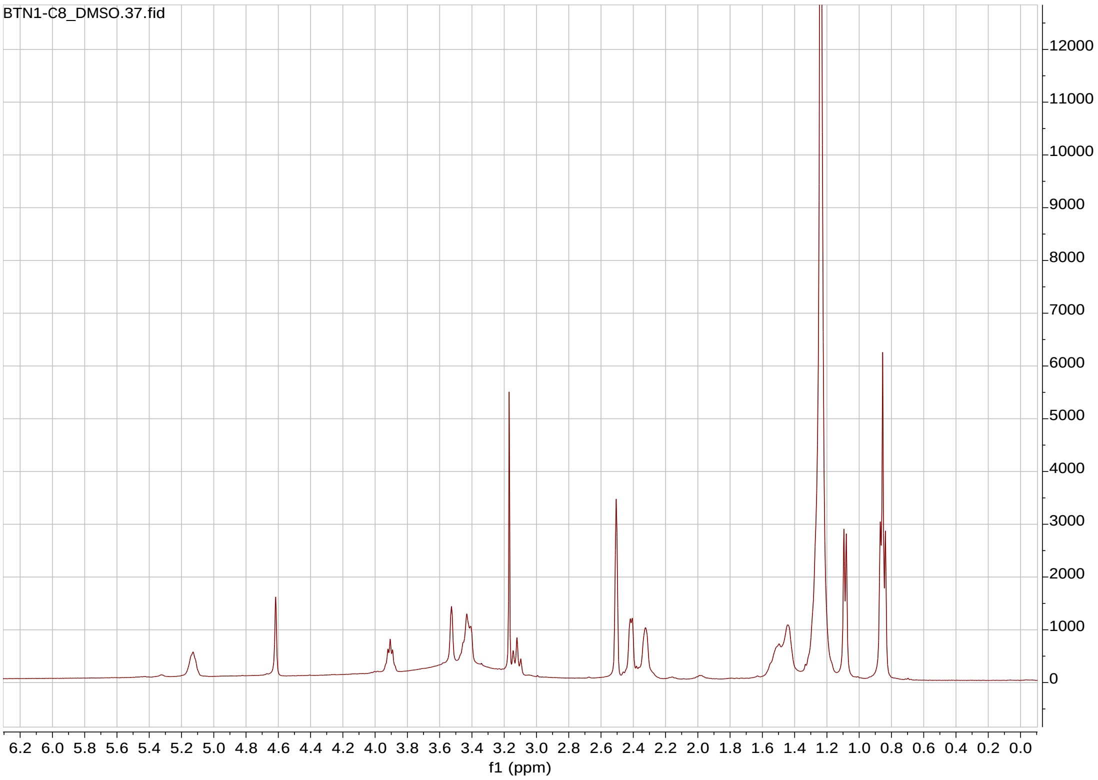

Figure S6. ^{1}H-NMR spectrum of compound **1** in DMSO-d6 at 400 MHz.

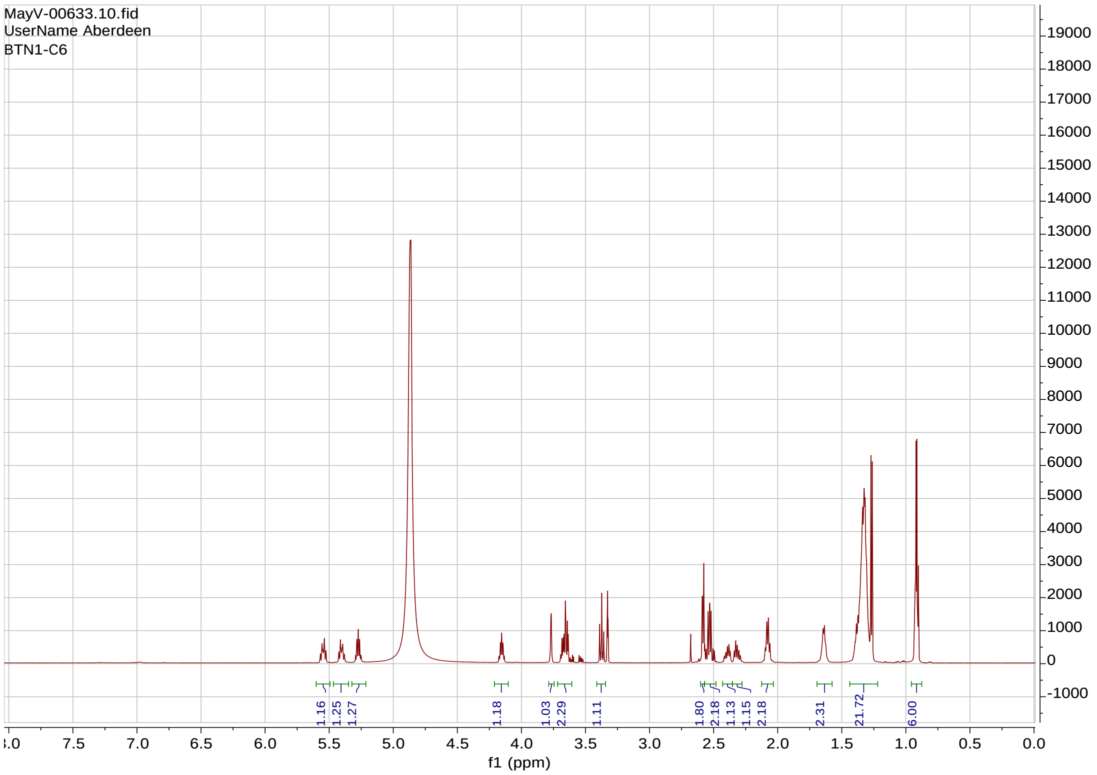

Figure S7. ^{1}H-NMR spectrum of compound **2** in CD_3OD at 600 MHz.

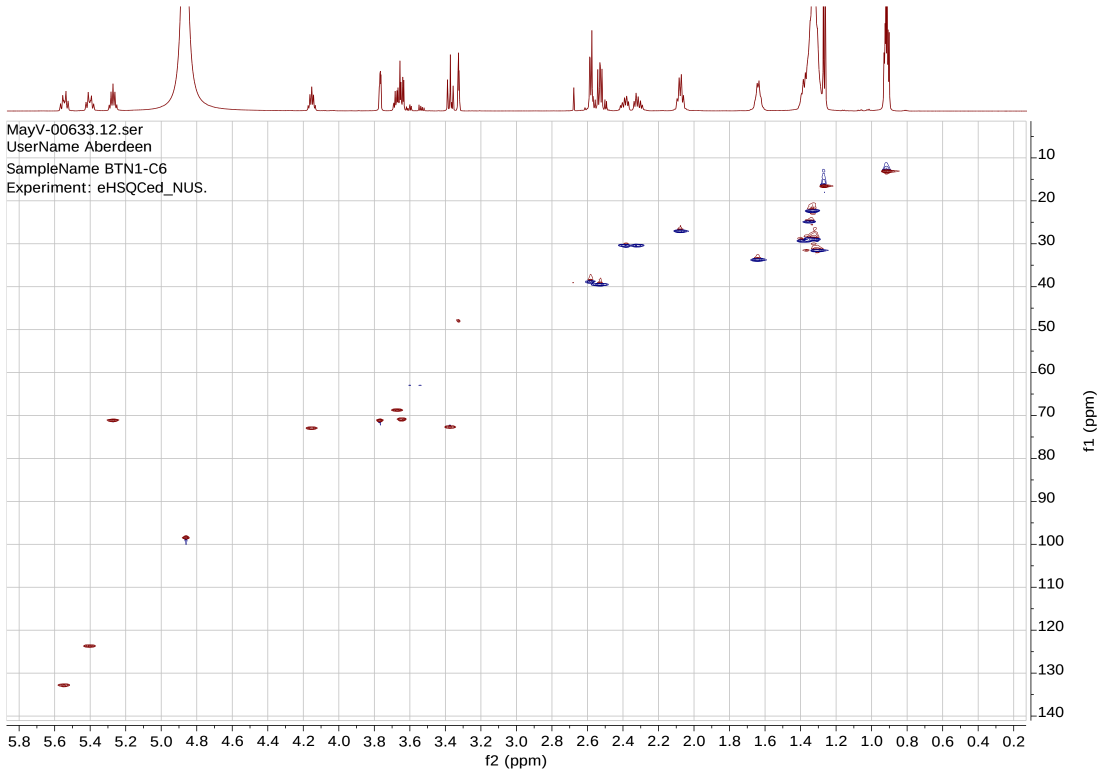

Figure S8. Edited-HSQC NMR spectrum of compound **2** in CD_3OD.

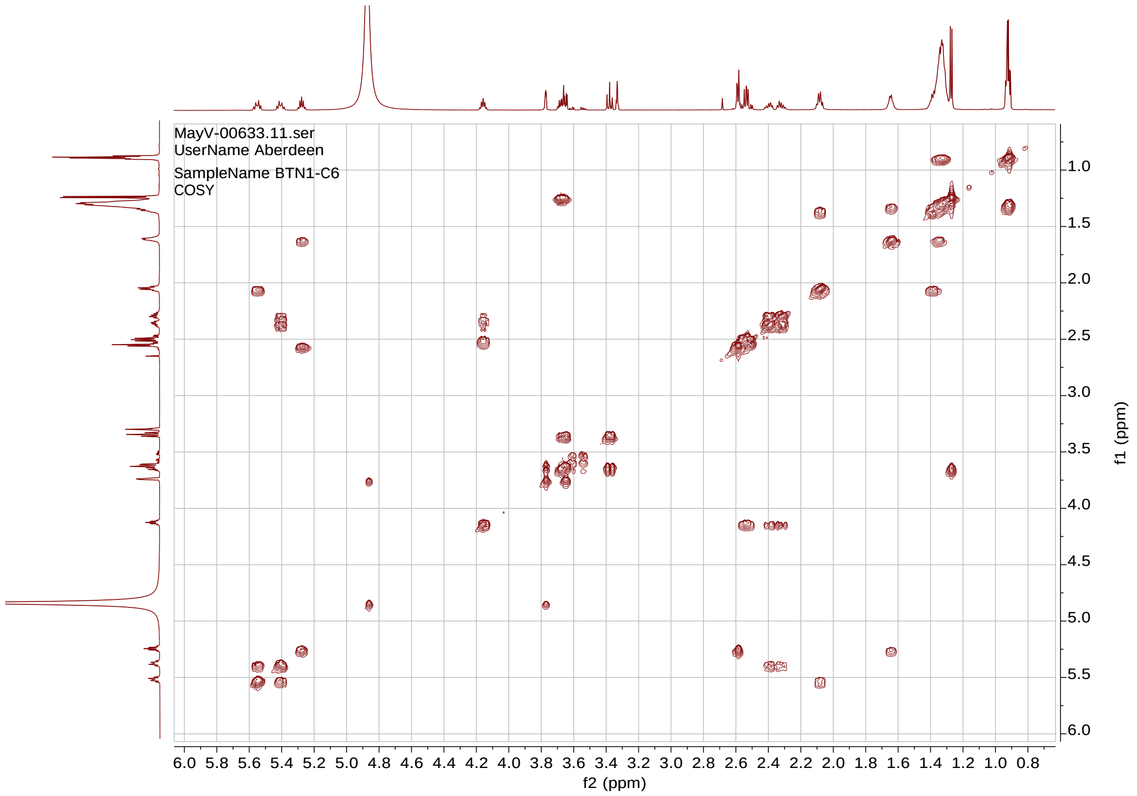

Figure S9. COSY NMR spectrum of compound **2** in CD_3OD.

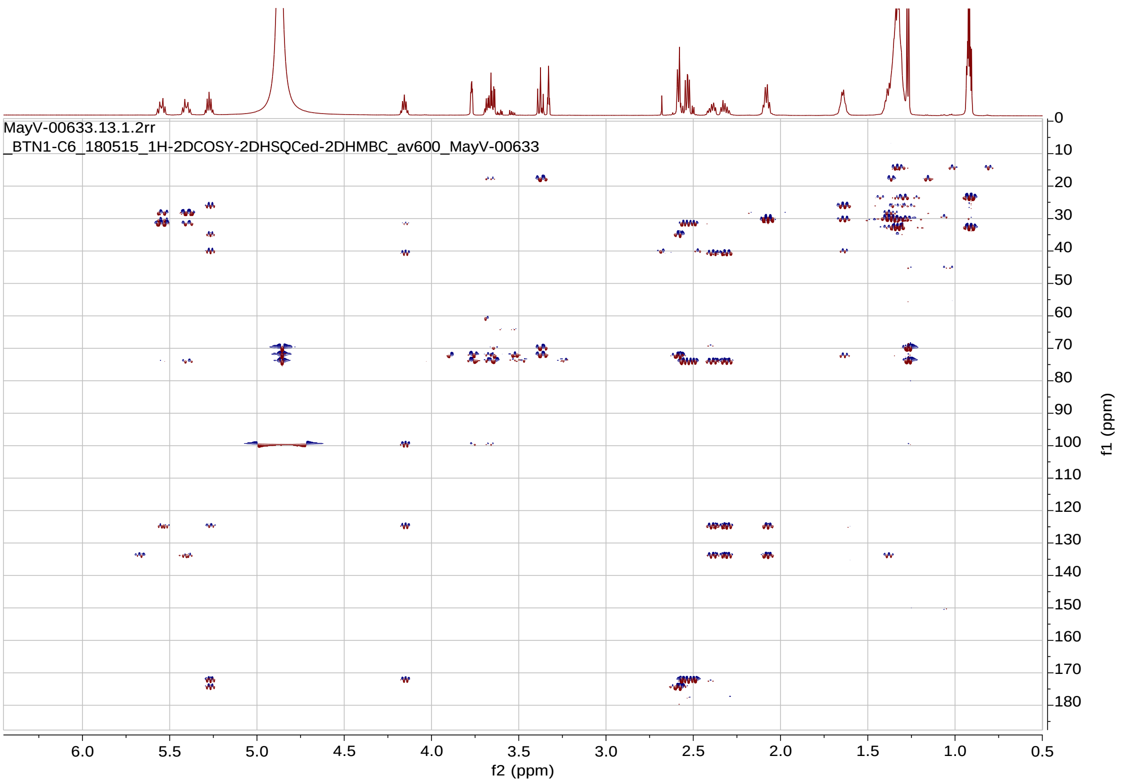

Figure S10. HMBC NMR spectrum of compound **2** in CD_3OD.

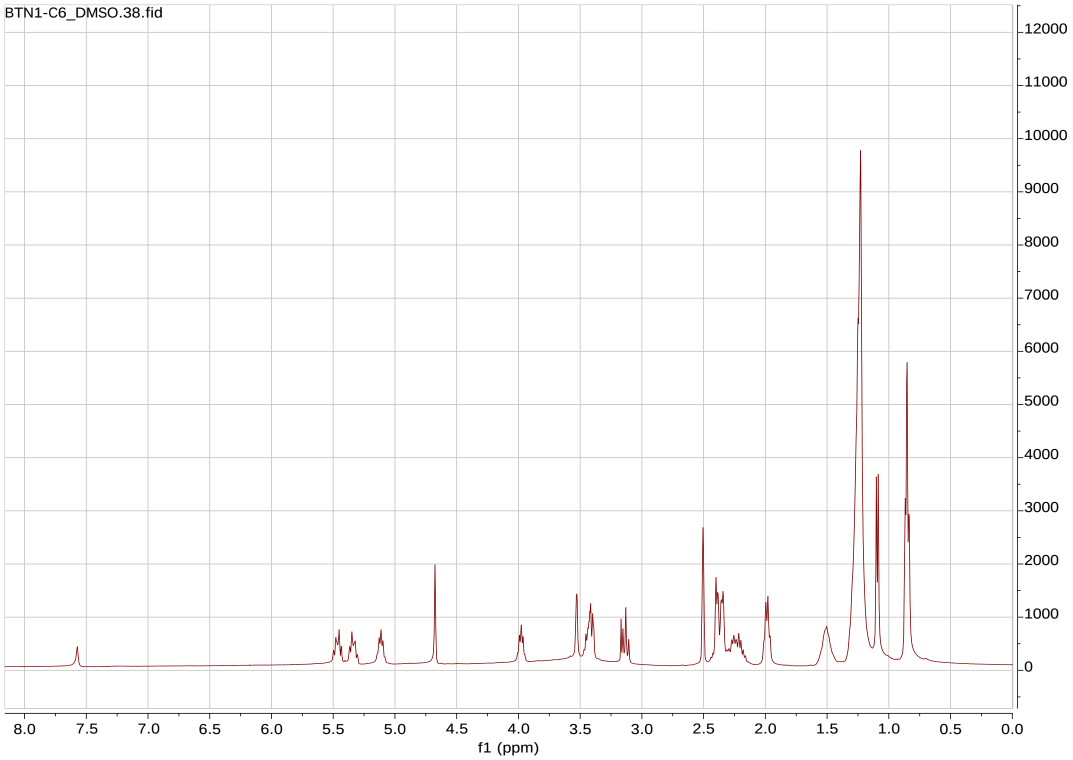

Figure S11. ^{1}H-NMR spectrum of compound **2** in DMSO-d6 at 400 MHz.

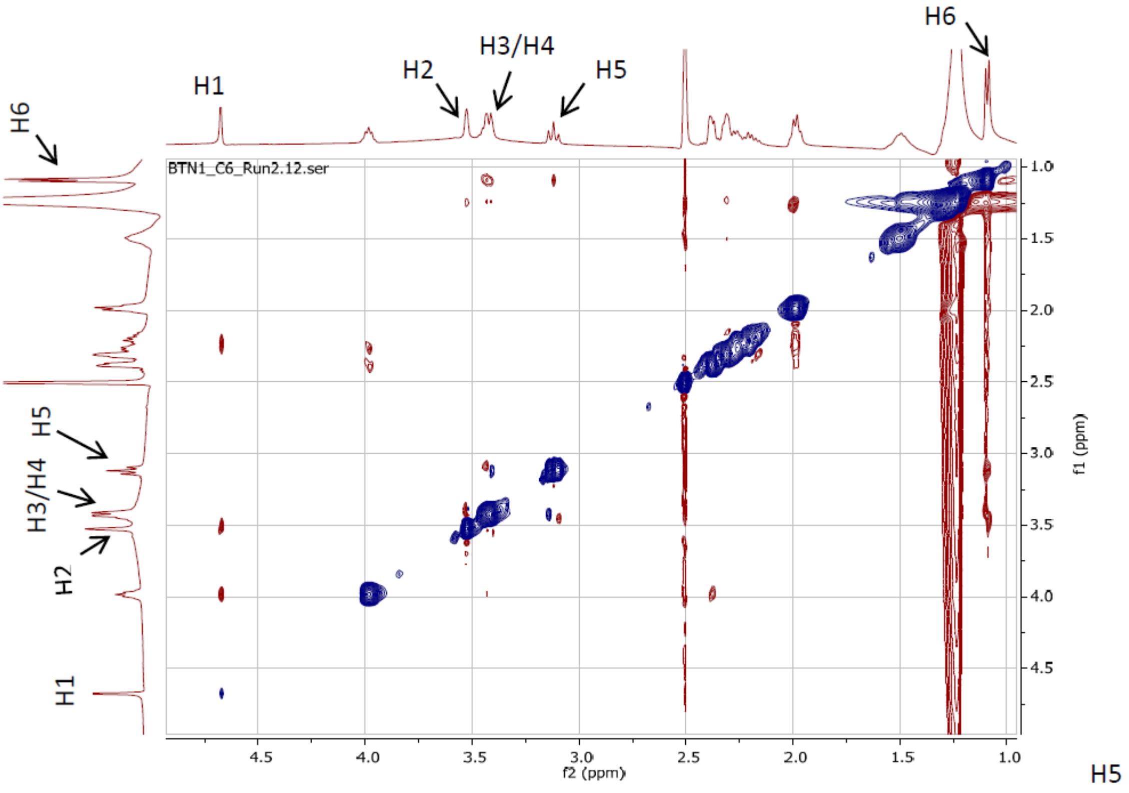

Figure S12. 2D ROESY spectrum of compound **2** in DMSO-d6 with Rhamnose protons labelled.

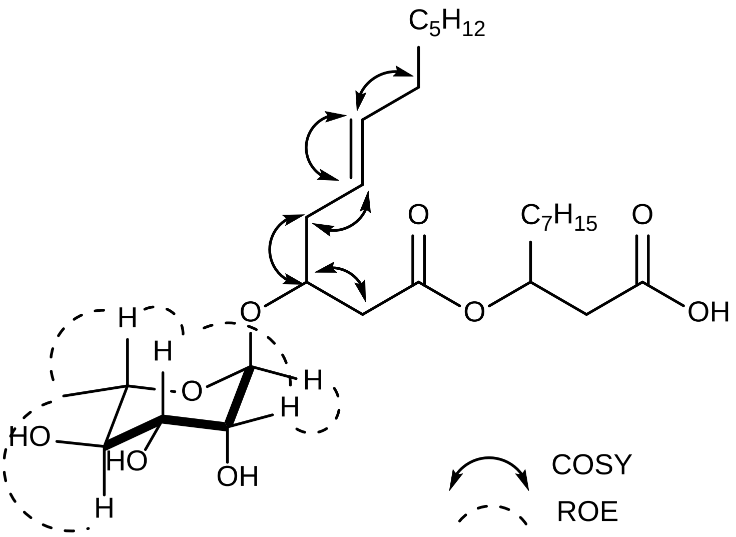

Figure S13. Compound **2** showing selected ROE correlations of the rhamnose moiety and COSY correlations showing position of unsaturation of the lipid chain.

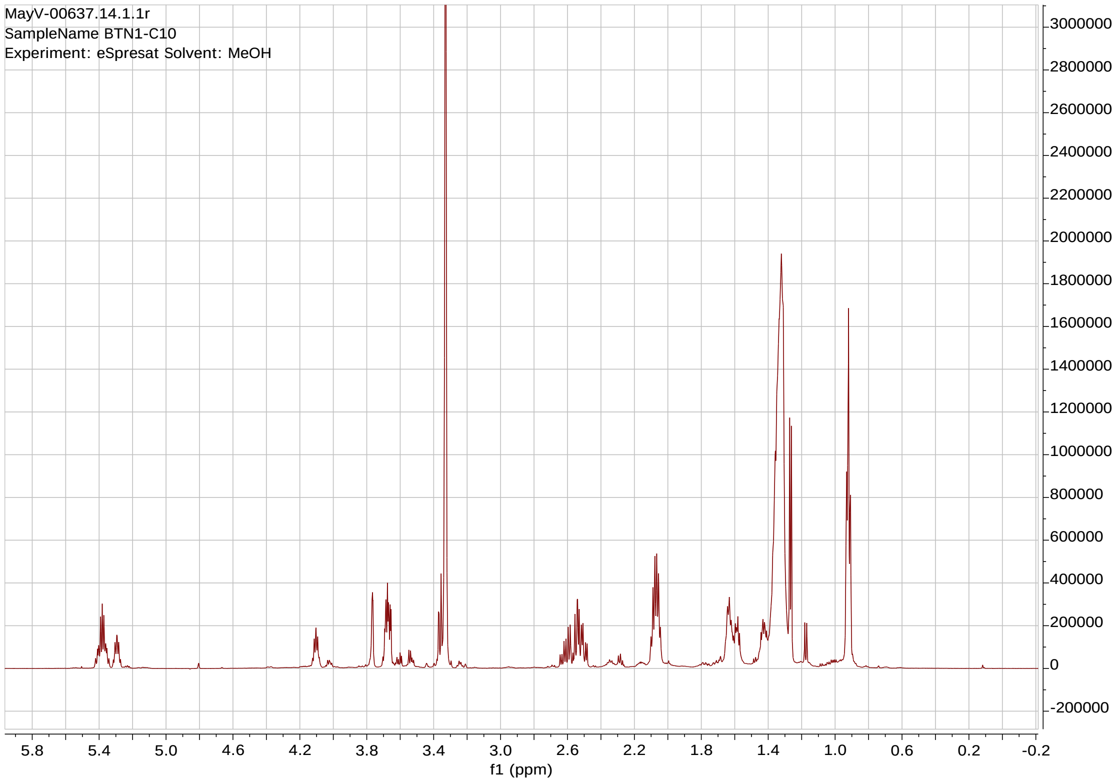

Figure S14. ^{1}H-NMR spectrum of compound **3** in CD_3OD at 600 MHz.

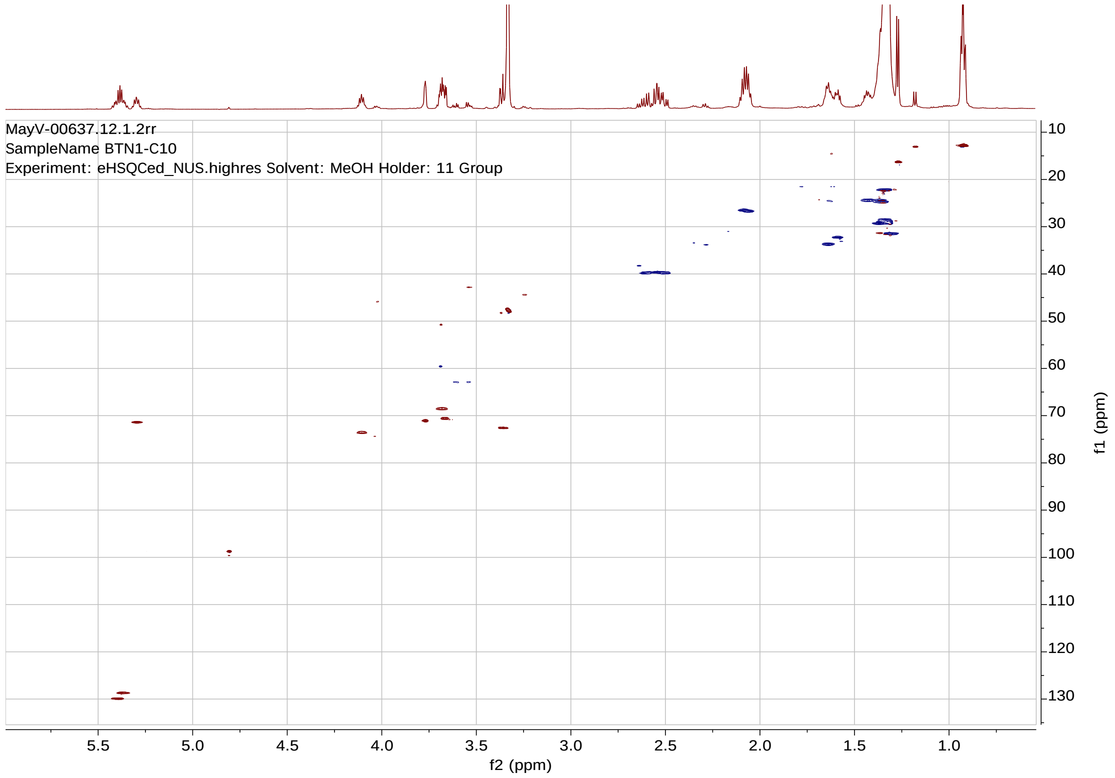

Figure S15. Edited-HSQC NMR spectrum of compound **3** in CD_3OD.

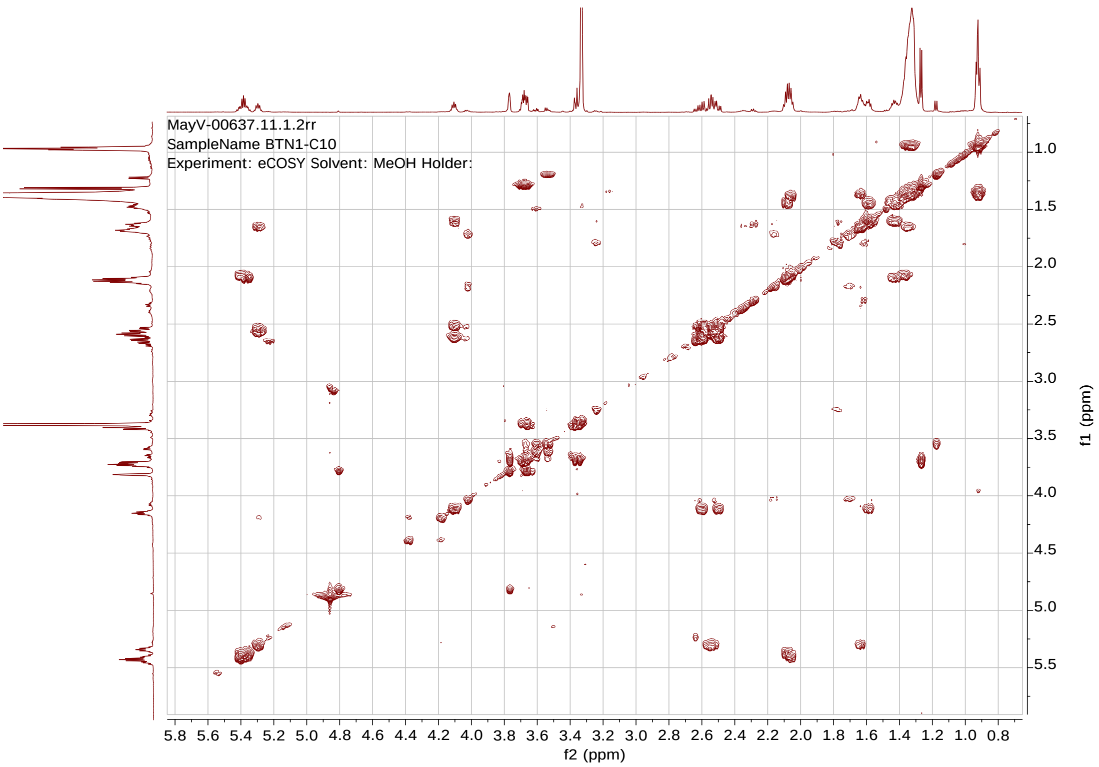

Figure S16. COSY NMR spectrum of compound **3** in CD_3OD.

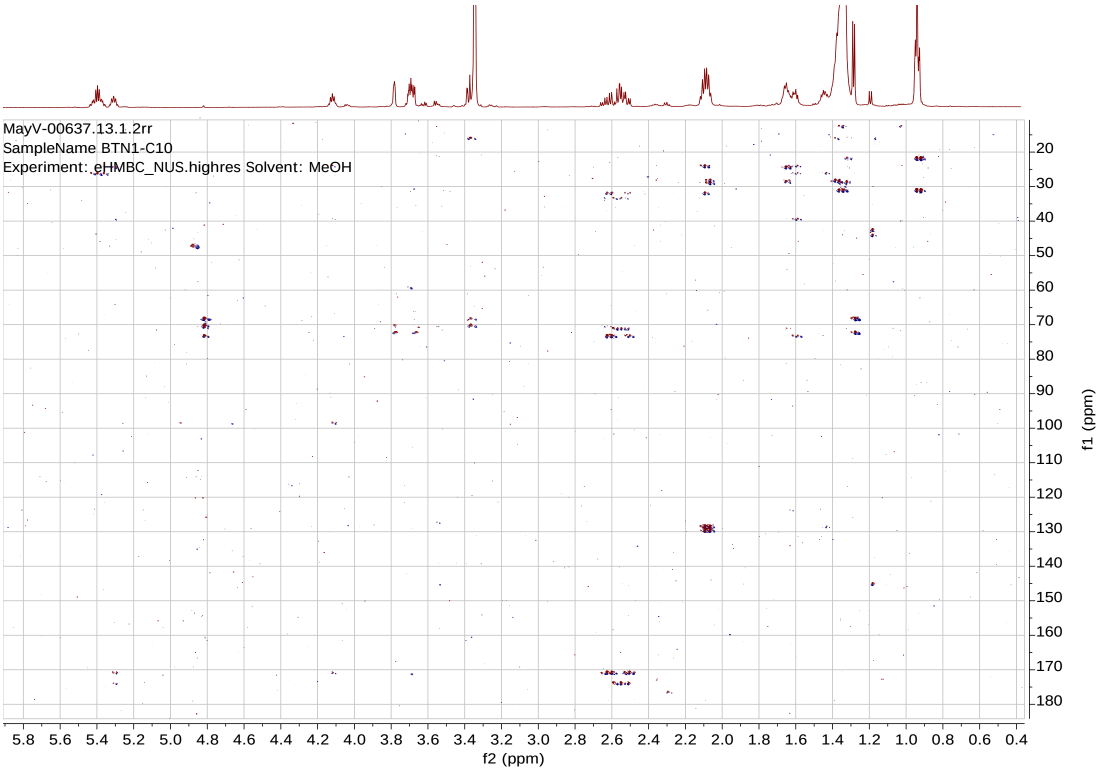

Figure S17. HMBC NMR spectrum of compound **3** in CD_3OD.

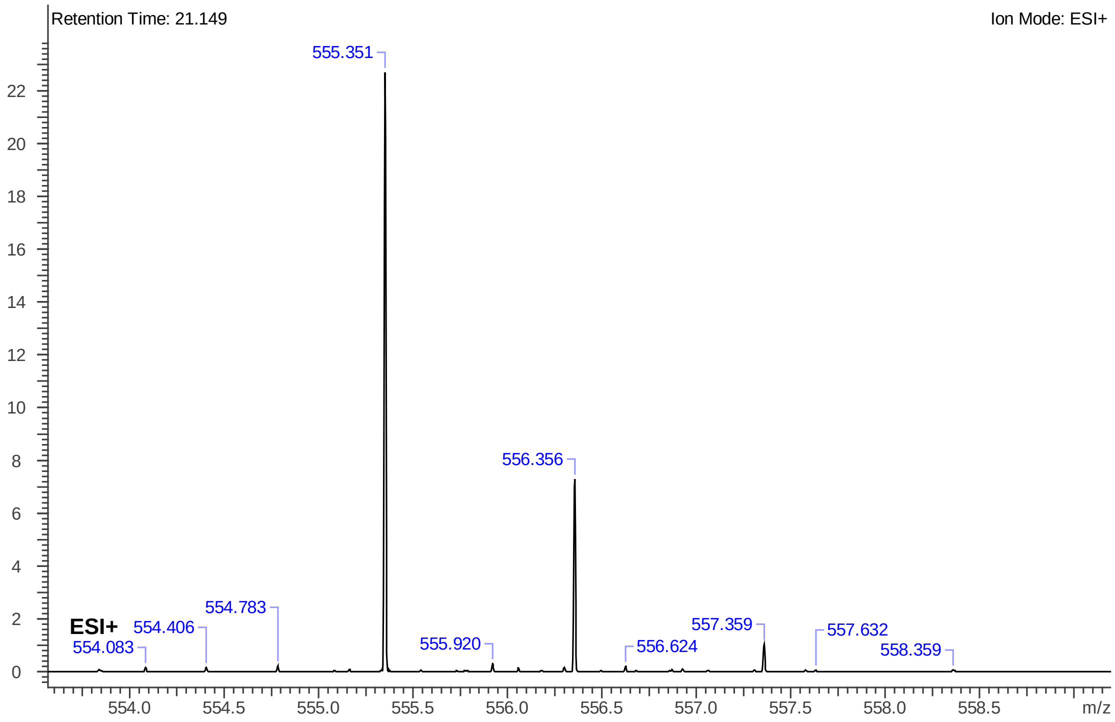

Figure S18. HRESIMS spectrum (M + Na)+ of compound **1**.

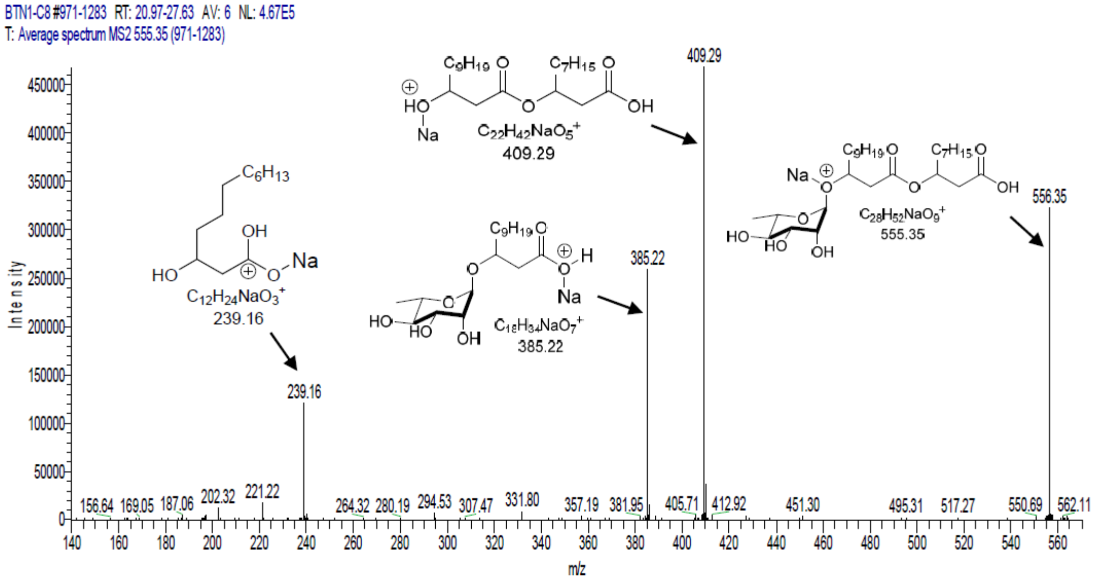

Figure S19. ESI MS/MS data of compound **1**.

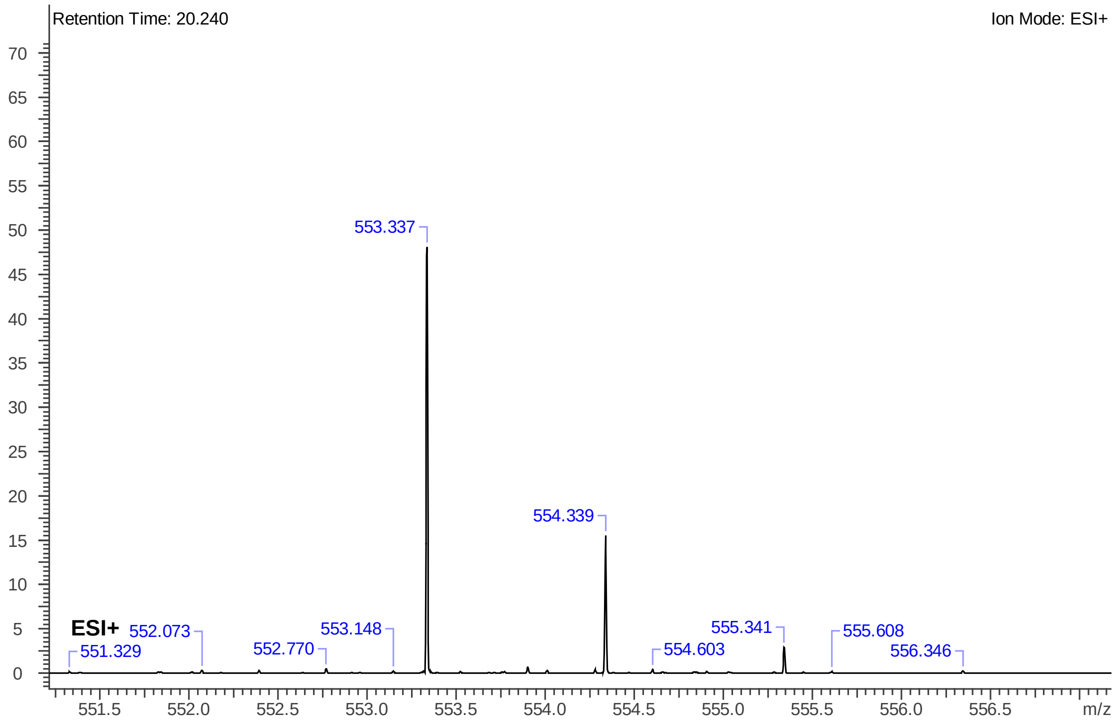

Figure S20. HRESIMS spectrum $(M + Na)^+$ of compound **2**.

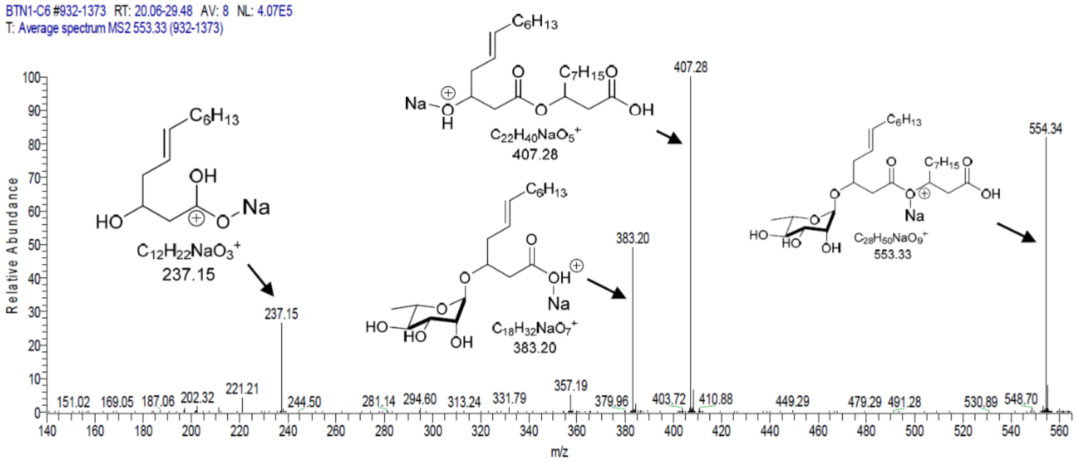

Figure S21. ESI MS/MS data of compound **2**.

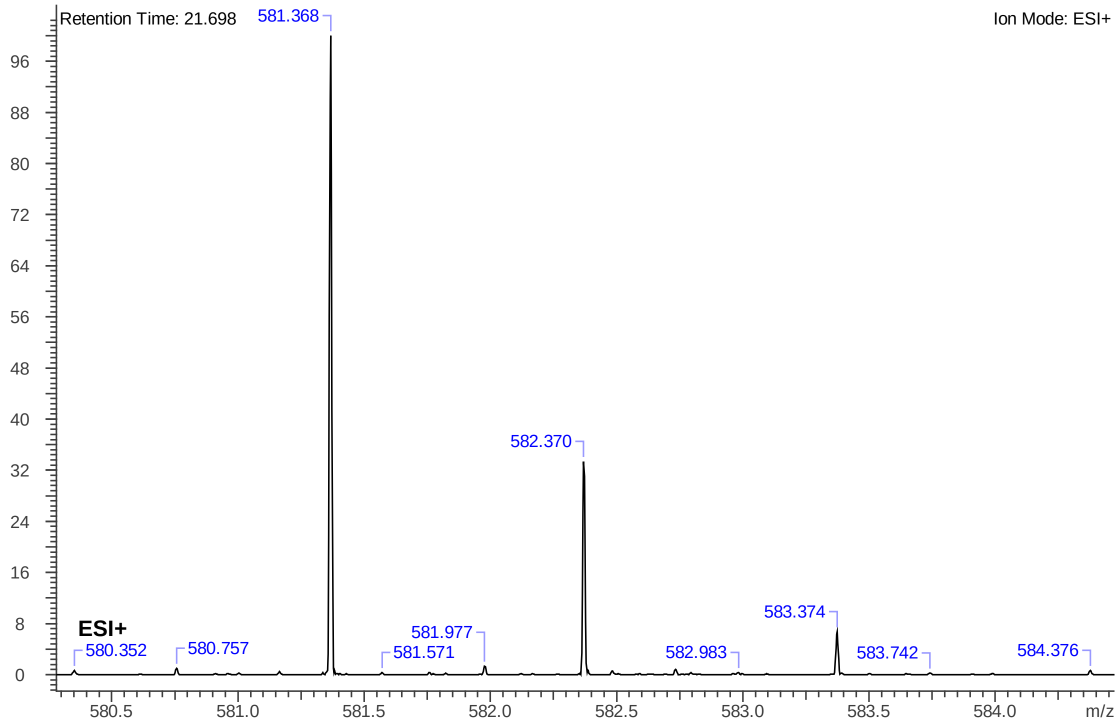

Figure S22. HRESIMS spectrum (M + Na)+ of compound **3**.

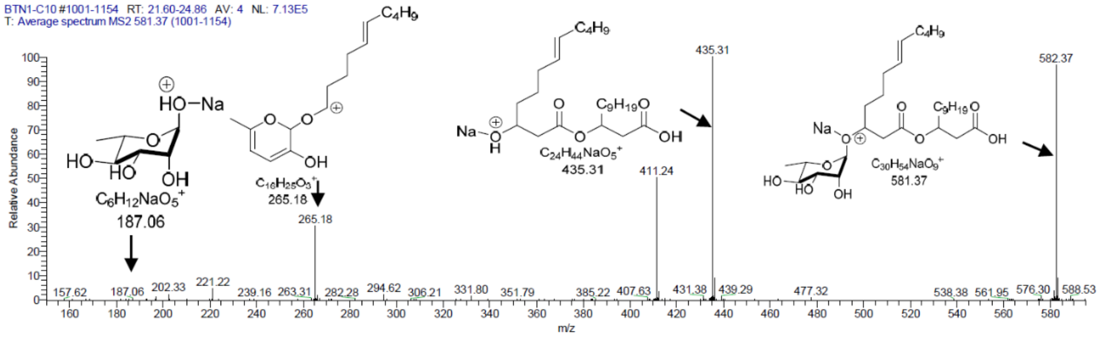

Figure S23. ESI MS/MS data for compound **3**.

Table S1. Cross- streaking experiments of BTN isolates against a wide panel of Bcc strains. The Bcc strains highlighted in red correspond to type strains.Symbols: +, growth; ±, reduced growth; -, no growth; C+, positive controls, *i.e.*, Bcc strains grown in the absence of the tester strain(s). Abbreviations: *Ps*, *Pseudomonas*; *Ar*, *Arthrobacter*; CF, Cystic Fibrosis; Env; environmental.

Bcc Species	Source	Strain	C+	*Ps*	*Psychrobacter*																	*Ar*
				BTN1	BTN2	BTN15	BTN3	BTN19	BTN20B	BTN21	BTN24	BTN23	BTN5	BTN12	BTN14	BTN17	BTN16	BTN18	BTN11	BTN13	BTN22	BTN4
B. cepacia	CF	**FCF 1**	+	-	-	-	-	-	-	-	-	-	-	-	-	-	-	-	-	-	-	-
	CF	**FCF 2**	+	-	-	-	-	-	-	-	-	-	-	-	-	-	-	-	-	-	-	-
	CF	**FCF3**	+	-	-	-	-	-	-	-	-	-	-	-	-	-	-	-	-	-	-	-
	Env	**LMG 2161**	+	-	-	-	-	±	-	-	-	-	-	-	-	-	-	-	-	-	-	-
B. multivorans	CF	**FCF 5**	+	-	-	-	-	-	-	-	-	-	-	-	-	-	-	-	-	-	-	-
	CF	**FCF 6**	+	-	-	-	-	±	-	-	-	-	-	-	-	-	-	-	-	-	-	-
	CF	**FCF 7**	+	-	-	-	-	±	-	-	-	-	-	-	-	-	-	-	-	-	-	-
	CF	**FCF 8**	+	-	-	-	-	±	-	-	-	-	-	-	-	-	-	-	-	-	-	-
	CF	**FCF 9**	+	-	-	-	-	-	-	-	-	-	-	-	-	-	-	-	-	-	-	-
	CF	**FCF 10**	+	-	-	-	-	-	-	-	-	-	-	-	-	-	-	-	-	-	-	-
	CF	**FCF 11**	+	-	-	-	-	-	-	-	-	-	-	-	-	-	-	-	-	-	-	-
	CF	**LMG 18822**	+	-	-	-	-	-	-	-	-	-	-	-	-	-	-	-	-	-	-	-
	Env	**LMG 17588**	+	-	-	-	-	-	-	-	-	-	-	-	-	-	-	-	-	-	-	-
B. cenocepacia	CF	**FCF 12**	+	-	-	-	-	-	-	-	-	-	-	-	-	-	-	-	-	-	-	-
	CF	**FCF 13**	+	-	-	-	-	-	-	-	-	-	-	-	-	-	-	-	-	-	-	-
	CF	**FCF 14**	+	-	-	-	-	-	-	-	-	-	-	-	-	-	-	-	-	-	-	-
	CF	**FCF 15**	+	-	-	-	-	-	-	-	-	-	-	-	-	-	-	-	-	-	-	-
	CF	**FCF 16**	+	-	-	-	-	-	-	-	-	-	-	-	-	-	-	-	-	-	-	-
	CF	**FCF 17**	+	-	-	-	-	-	-	-	-	-	-	-	-	-	-	-	-	-	-	-
	CF	**FCF 18**	+	-	-	-	-	-	-	-	-	-	-	-	-	-	-	-	-	-	-	-
	CF	**FCF 19**	+	±	-	-	±	-	-	-	-	±	-	±	±	-	±	-	±	-	-	±
	CF	**FCF 20**	+	-	-	-	-	-	-	-	-	-	-	-	-	-	-	-	-	-	-	-
	CF	**FCF 21**	+	-	-	-	-	-	-	-	-	-	-	-	-	-	-	-	-	-	-	-
	CF	**FCF 22**	+	-	-	±	-	-	±	±	-	-	±	-	-	-	-	-	-	-	-	-
	CF	**FCF 23**	+	-	-	-	-	-	-	-	-	-	-	-	-	-	-	-	-	-	-	-
	CF	**FCF 24**	+	-	-	-	-	-	-	-	-	-	-	-	-	-	-	-	-	-	-	-
	CF	**FCF 25**	+	±	-	-	-	-	-	-	-	-	-	-	-	-	-	-	-	-	±	-
	CF	**FCF 26**	+	-	-	-	-	-	-	-	-	-	-	-	-	-	-	-	-	-	-	-

	CF	**FCF 27**	+	-	-	-	-	-	-	-	-	-	-	-	-	-	-	-	-	-	-	-
	CF	**FCF 28**	+	-	-	-	-	-	-	-	-	-	-	-	-	-	-	-	-	-	-	-
	CF	**FCF 29**	+	-	-	-	-	-	-	-	-	-	-	-	-	-	-	-	-	-	-	-
	CF	**FCF 30**	+	-	-	-	-	-	-	-	-	-	-	-	-	-	-	-	-	-	-	-
	CF	**FCF 31**	+	-	-	-	-	-	-	-	-	-	-	-	-	-	-	-	-	-	-	-
	CF	**LMG 16654**	+	-	-	-	-	-	-	-	-	-	-	-	-	-	-	-	-	-	-	-
	CF	**C 5424**	+	-	-	-	-	-	-	-	-	-	-	-	-	-	-	-	-	-	-	-
	CF	**CEP 511**	+	-	-	-	-	-	-	-	-	-	-	-	-	-	-	-	-	-	-	-
	Env	**MVPC 1/16**	+	-	-	-	-	-	-	-	-	-	-	-	-	-	-	-	-	-	-	-
	Env	**MVPC 1/73**	+	-	-	-	-	-	-	-	-	±	-	-	-	-	-	-	-	-	-	-
	CF	**LMG 24506**	+	-	-	-	-	-	-	-	-	-	-	-	-	-	-	-	-	-	-	-
	Env	**LMG 19230**	+	-	-	-	-	-	-	-	-	-	-	-	-	-	-	-	-	-	-	-
	Env	**LMG 19240**	+	-	-	-	-	-	-	-	-	-	-	-	-	-	-	-	-	-	-	-
	CF	**FCF 32**	+	-	-	-	-	-	-	-	-	-	-	-	-	-	-	-	-	-	-	-
	CF	**FCF 33**	+	-	-	-	-	-	-	-	-	-	-	-	-	-	-	-	-	-	-	-
	CF	**FCF 34**	+	-	-	-	-	-	-	-	-	-	-	-	-	-	-	-	-	-	-	-
	CF	**FCF 36**	+	-	-	-	-	-	-	-	-	-	-	-	-	-	-	-	-	-	-	-
	CF	**FCF 37**	+	-	-	-	-	-	-	-	-	-	-	-	-	-	-	-	-	-	-	-
	CF	**FCF 38**	+	-	-	-	-	-	-	-	-	-	-	-	-	-	-	-	-	-	-	-
	CF	**FCF 39**	+	-	-	-	-	-	-	-	-	-	-	-	-	-	-	-	-	-	-	-
	CF	**LMG 21462**	+	-	-	-	-	-	-	-	-	-	-	-	-	-	-	-	-	-	-	-
B. stabilis	CF	**FCF 40**	+	-	-	-	-	-	-	-	-	-	-	-	-	-	-	-	-	-	-	-
	CF	**FCF 41**	+	-	-	-	-	-	-	-	-	-	-	-	-	-	-	-	-	-	-	-
B. vietnamensis	CF	**FCF 42**	+	-	-	-	-	-	-	-	-	-	-	-	-	-	-	-	-	-	-	-
B. dolosa	CF	**LMG 18941**	+	-	-	-	-	-	-	-	-	-	-	-	-	-	-	-	-	-	-	-
	CF	**LMG 18942**	+	-	-	-	-	-	-	-	-	-	-	-	-	-	-	-	-	-	-	-
B. ambifaria	Env	**MCI 7**	+	-	-	-	-	-	-	-	-	-	-	-	-	-	-	-	-	-	-	-
	CF	**LMG 19467**	+	-	-	-	-	-	-	-	-	-	-	-	-	-	-	-	-	-	-	-
B. anthina	Env	**LMG 16670**	+	-	-	-	-	-	-	-	-	-	-	-	-	-	-	-	-	-	-	-
	CF	**LMG 20983**	+	-	-	-	-	-	-	-	-	-	-	-	-	-	-	-	-	-	-	-
B. pyrrocinia	CF	**FCF 43**	+	-	-	-	-	-	-	-	-	-	-	-	-	-	-	-	-	-	-	-
	CF	**FCF 44**	+	-	-	-	-	-	-	-	-	-	-	-	-	-	-	-	-	-	-	-
	CF	**FCF 45**	+	-	-	-	-	-	-	-	-	-	-	-	-	-	-	-	-	-	-	-

	CF	**FCF 46**	+	-	-	-	-	-	-	-	-	-	-	-	-	-	-	-	-	-	-	-
	Env	**MVPC 1/26**	+	-	-	-	-	-	-	-	-	-	-	-	-	-	-	-	-	-	-	±
	Env	**MVPC 2/77**	+	-	-	-	-	-	-	-	-	±	-	-	-	-	-	-	-	-	-	-
	CF	**LMG 21824**	+	-	-	-	-	-	±	±	-	±	-	-	-	-	-	-	±	-	-	±
B. lata	CF	**LSED 4**	+	-	-	-	-	-	-	-	-	-	-	-	-	-	-	-	-	-	-	-
	Env	**LMG 6991**	+	-	-	-	-	±	±	±	-	±	-	-	-	-	±	-	-	-	-	±
B. ambifaria	Env	**LMG 19182**	+	-	-	-	-	-	-	-	-	-	-	-	-	-	-	-	±	-	-	-
B. anthina	Env	**LMG 20980**	+	-	-	-	-	-	-	-	-	-	-	-	-	-	-	-	-	-	-	-
B. cenocepacia	CF	**LMG 16656**	+	-	-	-	-	-	-	-	-	-	-	-	-	-	-	-	-	-	-	-
B. cepacia	Env	**LMG 1222**	+	-	-	-	-	-	-	-	-	-	-	-	-	-	-	-	-	-	-	-
B. contaminas	AI	**LMG 23361**	+	-	-	-	-	-	-	-	-	-	-	-	-	-	-	-	-	-	-	-
B. diffusa	CF	**LMG 24065**	+	-	-	-	-	-	-	-	-	-	-	-	-	-	-	-	-	-	-	-
B. dolosa	CF	**LMG 18943**	+	-	-	-	-	-	-	-	-	-	-	-	-	-	-	-	-	-	-	-
B. lata	Env	**LMG 22485**	+	-	-	-	-	-	-	-	-	-	-	-	-	-	-	-	-	-	-	-
B. latens	CF	**LMG 24064**	+	-	-	-	-	-	-	-	-	-	-	-	-	-	-	-	-	-	-	-
B. metallica	CF	**LMG 24068**	+	-	-	+	-	-	±	±	±	±	±	+	-	-	-	-	-	-	-	-
B. multivorans	CF	**LMG 13010**	+	-	-	±	-	-	-	-	-	-	-	-	-	-	-	-	-	-	-	-
B. pseudomultivorans	CF	**LMG 26883**	+	-	-	±	-	-	-	-	-	-	-	-	-	-	-	-	-	-	-	-
B. pyrrocinia	Env	**LMG 14191**	+	-	-	+	-	-	-	-	±	±	-	+	-	-	-	-	-	-	-	-
B. seminalis	CF	**LMG 24067**	+	-	-	-	-	-	-	-	-	-	-	-	-	-	-	-	-	-	-	-
B. stabilis	CF	**LMG 14294**	+	-	-	-	-	-	-	-	-	-	-	-	-	-	-	-	-	-	-	-
B. uborrensis	Env	**LMG 20358**	+	-	-	±	-	-	-	-	-	-	-	-	-	-	-	-	-	-	-	-
B. vietnamensis	Env	**LMG10929**	+	-	-	-	-	-	-	-	-	-	-	-	-	-	-	-	-	-	-	-
